# Synthesis, Characterization, and Reaction Studies of Pd(II) Tripeptide Complexes

**DOI:** 10.3390/molecules26175169

**Published:** 2021-08-26

**Authors:** Lindsey J. Monger, Dmitrii Razinkov, Ragnar Bjornsson, Sigridur G. Suman

**Affiliations:** 1 Science Institute, University of Iceland, Dunhagi 3, 107 Reykjavik, Iceland; ljm4@hi.is (L.J.M.); dmr2@hi.is (D.R.); 2 Max Planck Institute Chemical Energy Conversion, 45470 Mülheim an der Ruhr, Germany; ragnar.bjornsson@cec.mpg.de

**Keywords:** Pd(II), tripeptide, AspAlaGly, β-AspAlaGly, TrpAlaGly, DFT

## Abstract

The aqueous synthesis of Pd(II) complexes with alkylated tripeptides led to the hydrolysis of the peptides at low pH values and mixtures of complexed peptides were formed. A non-aqueous synthetic route allowed the formation and isolation of single products and their characterization. Pd(II) complexes with α-Asp(OR)AlaGly(OR), β-Asp(OR)AlaGly(OR), and TrpAlaGly(OR) (R = H or alkyl) as tri and tetradentate chelates were characterized. The tridentate coordination mode was accompanied by a fourth monodentate ligand that was shown to participate in both ligand exchange reactions and a direct removal to form the tetradentate coordination mode. The tetradentate coordination revealed a rare a hemi labile carbonyl goup coordination mode to Pd(II). Reactivity with small molecules such as ethylene, acids, formate, and episulfide was investigated. Under acidic conditions and in the presence of ethylene; acetaldehyde was formed. The Pd(II) is a soft Lewis acid and thiophilic and the complexes abstract sulfur from episulfide at apparent modest catalytic rates. The complexes adopt a square planar geometry according to a spectroscopic analysis and DFT calculations that were employed to evaluate the most energetically favorable coordination geometry and compared with the observed infrared and NMR data.

## 1. Introduction

Although the interactions of metal ions with various biological molecules, including peptides, have been well studied, the focus of these examinations is primarily under the purview of metallodrugs and metal toxicity [1,2,3,4,5]. However, modified biocompatible or biologically relevant frameworks are also applied to the mimic natural catalyst’s design [6].

Peptides coordinate to metal ions via the amide backbone, but to initiate coordination, a primary ligating group must be present [7,8,9,10]. For small peptides, this is facilitated by the presence of a side chain with a strong donor group or by the primary amine which tends to form five- or six-membered ring chelates through the amino, amide backbone or the carboxylate moiety [7]. Preferences for coordination vary between metal ions where Pd(II) has a high affinity for nitrogen and sulfur as soft donor atoms [11,12,13,14,15,16] and can initiate the deprotonation of amides, forming chelate at pH values below a pH of 2 [17,18].

**Figure 1:**
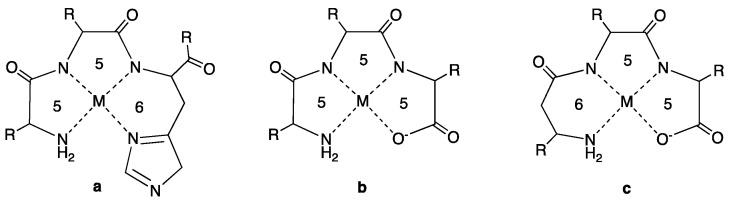
(**a**) ATCUN-motif, when M = Ni, Cu. (**b**) Coordination motifs for metal peptide complexes, M = Pd, Ni, or Cu. (**c**) Coordination motifs for metal peptide complexes with κ^4^[6,5,5] chelation.

Strong ligating side chain moieties also serve as anchor groups; histidine is a well-studied example [19,20,21]. The so-called ATCUN-motif (Amino Terminal Cu and Ni) forms strong coordinating ligands with histidine as the third amino residue for the N-terminus (as seen in Figure 1a). Innumerable possibilities of side chain combinations and modifications allow a variety of metal complexes to be synthesized with tunable geometries and electronic properties [8,10,20,22,23,24,25,26,27,28,29].

**Figure 2:**
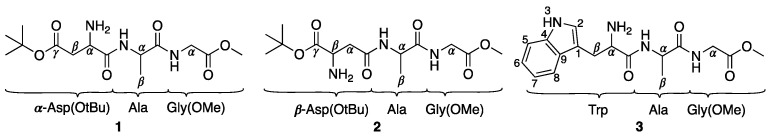
Tripeptide ligands **1**–**3**.

Bioinspired applications make use of modified biocompatible or biologically relevant frameworks to imitate the natural catalyst design [6]. Weakly coordinating chelates may be incorporated into the design of metal complexes to function as an “on-off” switch during catalysis. The modification of the carboxylic acid to form an ester could act in such a manner. A challenge encountered with this strategy is the ability of Pd(II) to catalyze ester hydrolysis [30]. In order to avoid this undesirable reaction, non-aqueous conditions were employed. In previous work we described the synthesis of three new tripeptide ligands and investigated the effects of pH on their coordination to [Pd(en)(H_2_O)]^2+^ [31]. The ligands were designed to form either κ^4^[5,5,5] (Figure 1b) or κ^4^[6,5,5] (Figure 1c) membered ring tetradentate ligands, allowing for an adjustable ligand design for ligands **1** and **2** (Figure 2). A similar approach employed histidine on the C-terminus of di- and tri- peptides with κ^4^[5,5,6] (Figure 1c)—type coordination [20,25]. Tryptophan was chosen for ligand **three** to impart organosolubility to an expected κ^4^[5,5,5] coordination. In addition to the primary amine and carboxylate C-terminal functional groups, indole complexation was reported through the secondary amine [32], the indole C2, [33,34], and the C3 carbon [35]. The tripeptides exhibited their maximum expected chelating ring sizes at the N-terminus and aqueous in situ studies confirmed it is possible to form complexes with κ^4^[*n*,5,5] (*n* = 8, 6, 5) chelates [31]. The molecular ions of the complexes were found and matched with their simulated isotope patterns in the ESI MS of the complexes. However, ester hydrolysis led to multiple species present in the reaction mixtures and non-isolable products.

This work outlines the synthesis of Pd(II) complexes of ligands **1**–**3**, and their characterization. The tetradentate complexes were subjected to reactivity studies to investigate their general properties.

## 2. Results

**Scheme 1:**
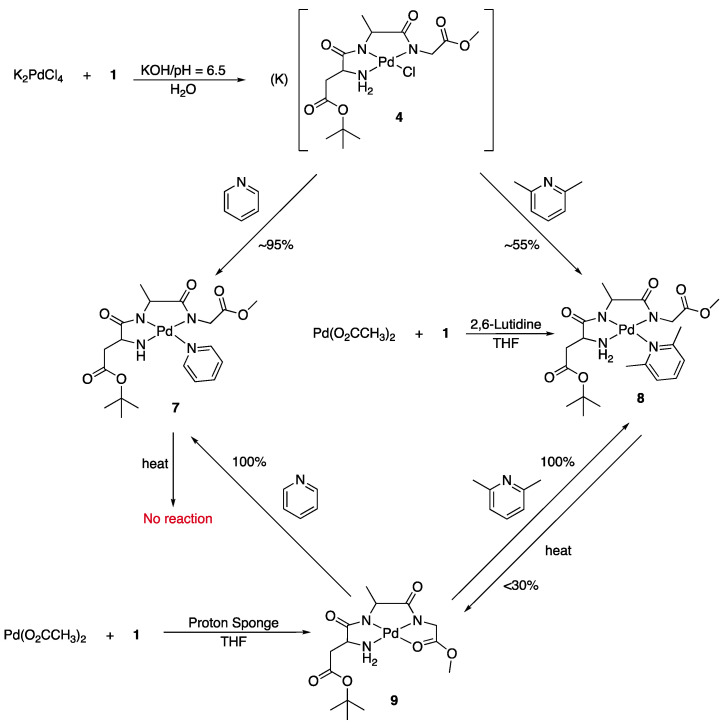
Scheme depicting synthetic routes and hemi-lability of complexes.

The ligand α-Asp(OtBu)AlaGly(OMe) (**1**) (Figure 2) was used to develop a synthetic strategy focused on achieving a neutral tetradentate Pd(II) complex. A summary of the synthetic routes employed is shown in Scheme 1. The procedure established was applied to ligands **2** and **3** (Figure 2).

### 2.1. Synthesis and Cation Exchange of Mono-Anionic Complexes ***4***–***6***

The coordination of tripeptide-**1** with Pd(II) and degree of hydrolysis are highly dependent on pH, rendering standard synthetic techniques ineffective [31]. Methods to establish an aqueous synthetic strategy to form neutral tetradentate Pd(II) complexes were explored [36]. In this strategy, K_2_PdCl_4_ was dissolved in water and added to an aqueous solution of **1**, then the pH was adjusted to ~6.5. The major product was the charged species K[Pd{α-Asp(O*^t^*Bu)AlaGly(OMe)}Cl], **4**. The composition of **4** was confirmed by mass spectrometry along with the presence of the Pd_2_L_2_Cl dimer. The coordination of **4** was κ^3^[NH_2_,N,N] as determined by spectroscopic data.

Complex **4** was synthesized with an improved reaction control by transitioning into a non-aqueous environment. Using Pd(CH_3_CN)_2_Cl_2_ as the starting complex and substituting KOH for two molar equivalents of triethylamine, resulted in the isolation of the complex (Et_3_NH)[Pd{α-Asp(O*^t^*Bu)AlaGly(OMe)}Cl], **5**. Pd(CH_3_CN)_2_Cl_2_ was isolated as the trans-isomer [37]. The acetonitrile had a stronger *trans* influence compared to Cl^−^ [38] and the presence of the chloro ligand in four may be owed to its slower displacement. The ^1^H-NMR resonances for different cationic salts **4** and **5** were identical, whereas the latter had an organic cation. Excess Et_3_NH^+^ proved difficult to remove, but this was resolved by performing a cation exchange reaction with PPh_4_^+^ to produce complex **six**, (Ph_4_P)[Pd{α-Asp(O*^t^*Bu)AlaGly(OMe)}Cl]. The cation exchange did not noticeably affect the complex ^1^H-NMR resonances. However, this led to the loss of yield and some decomposition. After the cation exchange, the integration of the ^1^H-NMR resonances for Ph_4_P^+^ and complex **6** confirmed **4**–**6** were mono-anionic, but had the same coordination geometry seen in Scheme 1 labeled **4**.

#### Ligand Exchange Reactions

To form the neutral complex (Scheme 1), reactions to exchange or remove the chloride were performed. Pyridine (Py) was added to the potassium salt (**4**) and the results monitored with NMR (Scheme 1, **7**). The spectrum showed new peaks in the 7–8 ppm region indicating coordination of pyridine. Integration confirmed the chloride was successfully replaced by pyridine. When seven was heated at 110 °C overnight, pyridine remained coordinated. In a second reaction, 2,6-lutidine (Lu) was added to **4** and the reaction monitored by ^1^H-NMR. Upon lutidine addition, the integration of its peaks in the NMR showed the incorporation of about 0.5–0.6 mol eq. of lutidine for the incomplete replacement of the chloride (Scheme 1, **8**).

A neutral palladium complex with coordinated lutidine was synthesized directly using Pd(OAc)_2_ and a two equivalence of 2,6-lutidine in dry THF (Scheme 1, **8**). Upon heating **8** overnight at 110 °C, tracking this reaction via ^1^H-NMR, a minor species started to form. Integration suggested the new species was less than 30% mol of the total yield where the changes to the resonances in the ^1^H-NMR of **8** upon heating were clearly observed. The formation of free Lu was also observed, confirming that lutidine’s labile coordination may be controlled with heat. This reaction was able to form the desired tetradentate complex Pd{α-Asp(O*^t^*Bu)AlaGly(OMe)} (**9**).

### 2.2. Synthesis of Neutral Complexes Tetradentate Pd(II) Complexes ***9***–***11***

**Figure 3:**
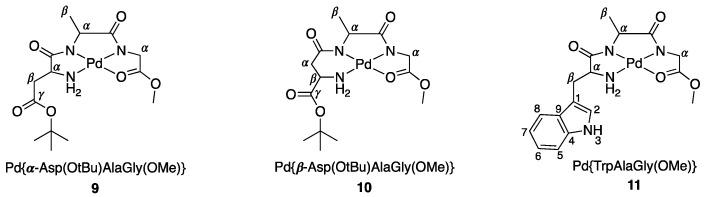
Tetradentate tripeptide Pd(II) complexes with coordinated ester carbonyl.

A non-aqueous strategy for the formation of **9** was developed, wherein **1** and a Proton Sponge (1,8-Bis(dimethylamino)naphthalene) were dissolved in dry THF, followed by the addition of Pd(OAc)_2_ to the solution, and stirring under argon. The Proton Sponge is a non-nucleophilic base which allows the last coordination site on the Pd(II) to remain open for the C-terminal ester to bind. The coordination of this complex is κ^4^[NH_2_,N,N,=O] with coordinated C-terminus methyl ester carbonyl as shown in Scheme 1. The composition of **9** was confirmed by mass spectrometry as well as an elemental analysis and fully characterized spectroscopically. This method established a synthetic approach of the neutral palladium tripeptide complex that was applied to ligands **2** and **3** to form **10** and **11**. (Figure 3). Complexes **10** and **11** were synthesized in good to high yields and fully characterized. These complexes were soluble in DMSO and DMF and were slightly soluble in acetonitrile and alcohols. However, they were insoluble in most other organic solvents.

The tripeptide-2 is an *iso*-peptide, meaning that it forms the peptide bond through the side chain carboxylic acid. When coordinated to a metal center via the N-terminal amine and the nearest amide, the peptide forms a 6-membered chelate. In an aqueous medium at neutral pH this system presented a competing coordination to the side chain carbonyl forming the bidentate 5-membered κ^2^[NH_2_,O] chelate instead of the κ^3^[NH_2_,N,N] amide backbone [31]. The κ^4^[NH_2_,N,N,O] chelated complex could be formed in basic aqueous conditions at a pH value of ~9. However, Pd(II) catalyzed hydrolysis at this pH value led to *t-*butyl and methyl ester hydrolysis of up to 35% and 65%. Thus, aqueous synthesis using two was not a viable option. However, utilizing the Proton Sponge as a base in dry THF yielded the κ^4^[NH_2_,N,N,=O] species, Pd{β-Asp(O*^t^*Bu)AlaGly(OMe)} (**10**), in good yields. 

Aqueous coordination studies of **3** with Pd(en)(H_2_O)^2+^ revealed that the tryptophan indole participated in coordination [31]. The ^1^H-NMR study revealed that at a low pH the NH_2_ coordinated as expected and chelation continued to the Ala amide. However, as the pH was increased to ~8 the κ^4^[NH_indole_,N,N,O] isomer was detected, forming an eight-membered ring. The study also showed that the methyl ester was hydrolyzed up to 75% under these conditions. Aqueous synthesis was, therefore, not attempted and **3** was complexed to Pd(OAc)_2_ in THF with the Proton Sponge. This yielded the κ^4^[NH_2_,N,N,=O] complex coordination mode (Figure 3, **11**).

#### Ligand Exchange Reactions

The coordinated ester carbonyl was expected to form a weak interaction with the metal center and act in a hemi-labile fashion. To test this, **9** was reacted with 1.5 mol eq of either Lu and Py and base coordination was monitored employing ^1^H-NMR. Both ligand exchange reactions went to completion immediately; see Scheme 1. The comparison of ligand exchange reactions of Lu and Py with **4** (Cl ligand) and **9** (carbonyl ester) proved the dissociation of the carbonyl ester oxygen was more rapid and gave higher yields. 

### 2.3. DFT Calculations

**Figure 4:**
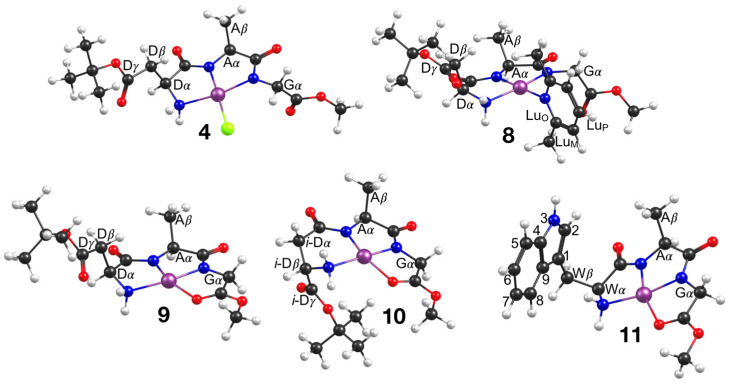
DFT-optimized structures of Pd(II) complexes **4**, **8**–**11**.

DFT calculations with a continuum solvation model were employed to determine the lowest energy structures in solution for complexes **4**–**11** which can be seen in Figure 4. (For cartesian coordinates, see Supplementary Materials). Several different binding modes and conformational possibilities were explored computationally. Due to the conformational flexibility of the tripeptide ligand, several conformers for each complex were available (primarily due different orientations of the Asp or Trp sidechains). The lowest energy conformer for each complex is shown in Figure 4 which was also used in the calculations of spectroscopic properties (Section 2.4). As the Pd(II) peptide complexes were preferably square planar, [NH_2_,N,N] coordination was expected, and DFT results revealed this.

In comparing the bond angles of the complexes, distinctive patterns were observed. Table 1 lists the calculated bond angles and bond lengths for the complexes. The tridentate complexes **4** and **8** more closely adhered to the ideal square planar geometry of 90°. In contrast, the tetradentate complexes **9** and **11** had a much smaller N_gly_-Pd-O_gly_ bite angle (~80°), increasing the NH_2_-Pd-O_gly_ coordination angle up to ~113 for **9** and **11**. Whereas the six-membered ring of **10** increased the NH_2_-Pd-N_ala_ bite angle from ~83° to 95°, and decreases the N_gly_-Pd-O_gly_ to 100°. The bond length assigned to complexes **9** and **11** were similar, whereas the six-membered chelate of **10** affected all metal coordination bond lengths.

### 2.4. Characterization

Selected ^1^H NMR and ^13^C NMR spectroscopic data are given in Table 2 and Table 3 respectively. Comparison of observed and calculated ^13^C NMR data is given in Table 4. Selected IR data is given in Table 5, and comparison of selected observed and calculated IR data is presented in Table 6.

Quantum chemical calculations at the DFT level were used to aid the spectral interpretation. To correctly assign coordination geometries, similarities and differences in the complexes’ ^13^C-NMR chemical shifts, and shifts in infrared frequencies of major bands were examined. Observed values were compared to calculated chemical shifts and vibrational frequencies of the DFT-optimized structural models (Table 4 and Table 6).

#### 2.4.1. H- and ^13^C-NMR Spectroscopy

The ^1^H and ^13^C spectra of **4**–**11** were obtained in DMSO-*d*_6_ and the resonances were assigned with the aid of COSY and HSQC measurements (Figures S1–S6). DMSO-*d*_6_ was selected since it allowed the monitoring of the NH_2_ protons, and the complexes were highly soluble.

**Figure 5:**
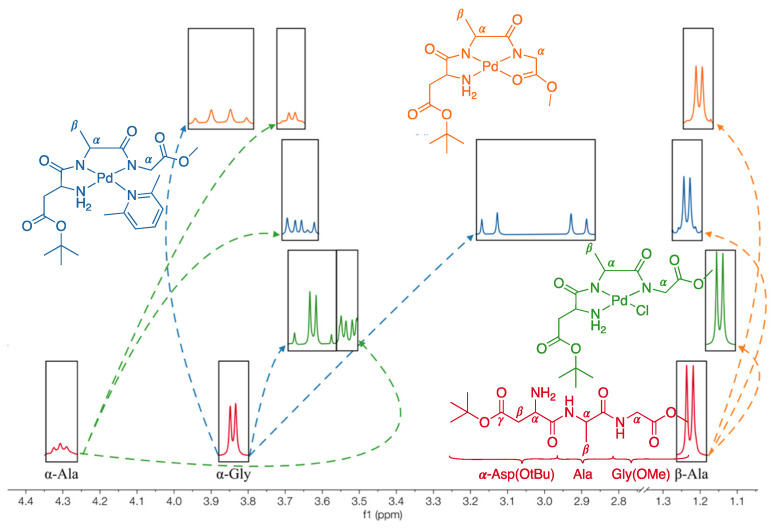
Progression of select ^1^H-NMR resonances of **1** (red) to **4** (green), **8** (blue), and **9** (orange).

The coordinated amine/amide backbone was a commonality of **4**–**11**; this coordination was established in the proton NMR (Table 2) with the appearance of the amino protons as two triplets between ~5.0 and 3.8 ppm, while the amide protons, located ~8.40–8.10 ppm, disappeared. Generally, compared to the free ligands **1**–**3** (Table S1), the α-C protons shifted upfield, whereas Ala α-C protons tended to shift the most and had the largest range of movement, as seen in Figure 5. This behavior was expected considering the Asp amide was trans to the fourth coordination site, counting clockwise from the anchoring amine group; thus, the magnetic environment of these protons changed more significantly.

The ^1^H-NMR resonances for **4** generally shifted upfield the most compared to **7**–**9**, presumably since this is the only negatively charged species, and the chloride’s π-donation to palladium effectively increased shielding around the ligand. The coordination of pyridine to **4** resulted in the amino, Asp, and Ala α-C protons all shifting downfield (Table 2, **7**), which is explained by pyridine π-back-bonding, resulting in metal deshielding. However, the Gly α-C and methyl ester protons shifted further upfield; one explanation for this is π interactions with neighboring pyridine. Heating **7** overnight at 110 °C did not show any observable changes in the NMR. The complex with the coordinated Lu (**8**) had slightly different spectroscopic behaviors compared to **7**. Notably, a large upfield shift of the NH_2_ protons (Δδ 0.20 and 0.23 ppm). In **7**, the Gly protons experienced the largest shielding effects of the series, appearing with a large coupling constant of ~96 Hz with resonances at 3.14, and 2.91 ppm (Figure 5). This is further evidence of a π interaction between the lutidine and the glycine moiety. Upon heating **8** overnight at 110 °C, a new species was observed, where the Lu was no longer coordinated. This new species matched the spectrum of **9**.

Support for the coordination mode of **9** was established in part by examining the ^1^H-NMR. The appearance of the NH_2_ protons at 4.76 and 5.03 ppm established the amine coordination. The Asp α-C proton appeared at about the same resonances as in one. However, the Ala α-C protons experienced a large upfield shift, supporting the Asp amide coordination. The Gly protons only shifted by 0.03 ppm compared to one, but the Gly coupling pattern changed from a doublet to a quadruplet, signifying Ala amide coordination as seen in Figure 5. The small change in resonances is explained with carbonyl oxygen coordination. A carbonyl group was a π-acceptor ligand, deshielding the metal. An argument for a coordinated ester via carboxylate oxygen was not supported, given the small change in the methyl ester chemical shift.

The ^1^H-NMR spectrum of **10** was poorly resolved owing to the lability of the six-membered ring. However, important information regarding coordination geometry was gleaned from the 2D-NMR coupling patterns and discernible resonance positions. Elevated temperature ^1^H-NMR experiments (330 K) were performed to improve the spectral resolution. Coordination of the amine was evidenced by the NH_2_ resonances at 4.63 and 4.48 ppm. Side chain methylene protons of the N-terminal amino acid (Asp or Trp) were a characteristic feature of the ligands **1**–**3**. These protons were diastereotopic and when recorded in DMSO-*d*_6_, appeared as a pair of doublet of doublets. This is because they were in different magnetic environments with different vicinal coupling. For 4–9, these protons were outside a chelate ring and showed similar coupling constants as the ligands. However, 10 (Table 2) enclosed the methylene protons in a six-membered ring and they became an apparent quadruplet. Additionally, a large upfield shift of the α-C protons for Asp (Δδ 0.46 ppm), Ala (Δδ 0.42 ppm), and Gly (Δδ 0.21 ppm) confirmed the amine/amide backbone chelation. 

In **11**, the appearance of the NH_2_ protons at 4.96 and 4.24 ppm confirmed the N-terminus amine coordination, whereas the indole resonances were relatively unchanged, suggesting the N-indole amine remained uncoordinated. Ala (Δδ 0.56 ppm) and Trp (Δδ 0.11 ppm) α-C protons shifted downfield and were in agreement with **9** and **10**. 

The ^13^C NMR spectrum collected for the ligands **1**–**3** (Table S2) and complexes **4, 8–11** (Table 3) were tabulated and compared. Assignments for the carbon spectrum were aided by the 2D-NMR technique HSQC. As expected, all α-carbons shifted downfield, while Ala α-carbons underwent the largest downfield shifts and had the broadest range.

The most significant and difficult ^13^C assignments belonged to the carbonyl carbons. In order to confidently assign these signals, DFT calculations were used to elucidate coordination modes and to calculate the ^13^C chemical shifts. The calculations were based on the optimized geometries depicted in Figure 2. Table 4 shows a comparison of observed and calculated carbonyl ^13^C chemical shifts, which were adjusted for systematic differences in shielding values. For **4** and **8,** there was a decent agreement between the observed and calculated values for Gly and Asp esters, and Asp amides. However, the Ala amide showed a stronger deshielding than calculations suggested. The coordination of the ester carbonyl (C=O_Gly_), observed in **9** and **11**, was accompanied by a large downfield shift from ~170 (**1**–**3**) to ~186 ppm, which was consistent with reported values [39]. Due to the lability of **10**, this peak was not observed, even in the high temperature NMR experiment. 

#### 2.4.2. Infrared Spectroscopy

Infrared spectroscopic data were collected in κBr discs and are detailed for ligands **1**–**3** and for complexes **4**, **8**–**11** in Table 5 and Supplementary Materials Figure S7. Vibrational modes for the ligands and complexes were assigned with the aid of DFT calculations, based on relative value sets. Comparative values for observed and calculated carbonyl stretching bands for **9**–**11** are presented in Table 6.

For **4**, **8**–**11**, the low energy shift of symmetric and asymmetric stretching modes for NH_2_ to energies typical of coordinated N_amino_, ~3250–3100 cm^−1^, [40] confirmed amine coordination, and the Amide I bands, which were mostly ν(C-N) shifted to low energy and combined with the Amide II bands; this supports the amine/amide backbone chelation. Additionally, palladium to nitrogen vibrational energies appeared and were consistent with reported values [40,41,42,43].

For **4** and **8**, the methyl ester carbonyl band was still present at ~1750 cm^−1^, indicating that the carbonyl was not coordinated. Lutidine coordination was confirmed for **8** with the appearance of an aromatic ν(C=C) band at 1471 cm^−1^ (free Lu 1481 cm^−1^) and by the shift of the out-of-plane CH band to a higher frequency (787 cm^−1^) compared to that of the free ligand (770 cm^−1^) [40]. Pd-Cl vibrations appeared at far-IR frequencies and were not observable. Compared to **4** and **8**, the spectrum for **9** had a strong distinct band at 1541 cm^−1^, as seen in Table 6. Palladium complexes with similar ester coordination were synthesized and were reported to exhibit a large shift of the ester carbonyl stretching frequency to lower energies [39,44] upon coordination. This indicates that the methyl ester carbonyl oxygen was coordinated to Pd, resulting in a lower energy shift of ~200 cm^−1^. This assignment is supported by the calculated relative vibrational frequencies for this complex; a similar band was present in the spectra collected for **10** and **11**, suggesting **9**–**11** had methyl ester carbonyl coordination. In addition, the methyl ester ν(C-O) for **9**–**11** appeared to have a similar low energy shift of ~7 cm^−1^.

#### 2.4.3. Mass Spectrometry

MS spectra were obtained using ESI-MS in the negative ion scan for anions and the positive ion scan for neutral or positive ions (Supplementary Materials Figures S1b, S3b, S4c, S5c and S6c). The found ion peaks were simulated with expected isotope patterns to confirm the composition of the compound found compared to the expected composition. Complexes **4**–**6** were obtained in the negative ion mode where the major isotope pattern observed was the [Pd{Asp(O*^t^*Bu)AlaGly(OMe)}Cl]^−^ ion, confirming these complexes were the anionic chloride species. The major isotope pattern for the neutral complexes was the [M + Na^+^]^+^ species. The isolated compounds showed an excellent match with less than 2 ppm variation in simulated vs. found values.

### 2.5. Reactivity Studies

#### 2.5.1. Sulfur Reagents

Palladium is a soft Lewis acid metal and is, therefore, thiophilic. Reacting palladium tripeptide complexes with episulfides could be a way to open coordination on the tetradentate complex and activate the metal. The tetracoordinated Pd(II) complex with NS_3_ donor sets was reported able to catalytically cleave a C(sp_2_)–I bond to form a Pd–I bond despite coordination saturation [45], suggesting a single coordination site could be sufficient for a sulfur transfer reaction or organosulfur polymerization. For **9**–**11**, after the initial episulfide coordination, several outcomes were conceivably possible. Either (a) the sulfur could coordinate in an oxidative addition reaction and form a palladium(IV) sulfur complex, expelling cyclohexene; (b) Pd(II) would react with the sulfur substrates to coordinate sulfur and a second episulfide could react with the coordinated episulfide to form a polythioether; (c) a catalytic sulfur transfer could occur, expel sulfur as S_n_ and Pd(II) coordinate to the resulting olefin [46].

**Scheme 2:**
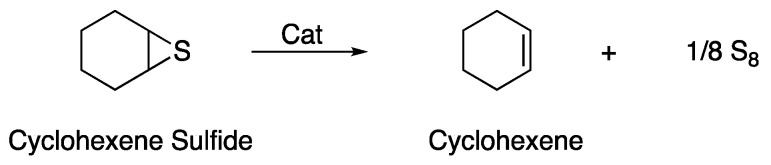
Catalytic conversion of cyclohexene sulfide to cyclohexene.

**Figure 6:**
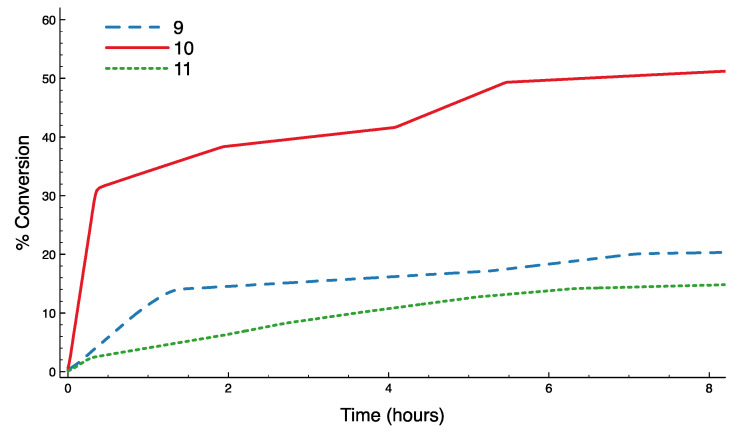
Percent catalytic conversion of cyclohexene sulfide to cyclohexene with 3 mol% complexes **9**, **10**, and **11**, monitored via ^1^H-NMR spectroscopy.

Cyclohexene sulfide was reacted with complexes **9**–**11** and the results were monitored by ^1^H-NMR to the determine conversion of thiirane to alkene in an overall reaction seen in Scheme 2 (Figures S8 and S9). The percent conversion to cyclohexene is detailed in Figure 6 as a function of time. 

For **9**, a quick conversion of cyclohexene sulfide to cyclohexene reached up to 20%, which slowed down after ~30% cyclohexene formation was observed. The deactivation of the catalyst was confirmed at ~30 h. Complex **10** showed a much higher conversion to cyclohexene, with conversion reaching >50% after 24 h before the deactivation of the catalyst. The lowest conversion was seen with **11**, where initial conversion was slow and plateaued at ~15%. For the most relevant industrial catalysts, the turnover frequency (TOF, mol product/mol catalyst x time) was in the range 36–360,000 h^−1^ [47]. The best TOF for **9**–**11** was observed for **10** (3 mol %) with a value of 2.59 h^−1^. Post-reaction work-up of the ^1^H-NMR sample showed that the ligand was partially demetallized. The sample from the catalytic reaction of **10** with cyclohexene sulfide was examined post-reaction. The solution was filtered, taken to dryness under reduced pressure, and ^1^H-NMR was recorded. The NMR spectrum of the post-catalytic solution exhibited resonances for free-ligand **2**, confirming that **10** did not remain intact (Supplementary Materials Figures S8 and S9). Solubility based separation of the ligand and the viscous material that formed was performed. The ^1^H-NMR of the viscous material was in agreement with the polythioether formation [46]. The reactions with episulfides, therefore, indicated that both alkene and polythioether may form; although these results were preliminary. It was noted that the cyclohexene formed did not coordinate to the metal center in these reactions.

To ensure the reaction conversion was not spontaneous, a blank sample maintaining same conditions but omitting the complex was run. That reaction resulted in no identifiable alkene formation for commensurate times as the runs, including complexes.

#### 2.5.2. Olefins

Palladium is known to coordinate to ethylene in an η^2^ fashion [48]. The Pd(II) complex with a tridentate ligand and a hemilabile methoxy ligand was shown to catalyze ethylene oligomerization [49]. The intention here was to study the availability of the 4th coordination site for reactivity and hemi-labile coordination, by either removing the chloride or lutidine ligand or by opening the chelate on the C-terminus ester carbonyl, thereby accessing the 4th coordination site that could serve as a reaction site. 

To examine whether these complexes are good candidates for reactions with olefins; ethylene reactivity studies were performed and monitored with ^1^H-NMR spectroscopy (Figure S10). The general procedure was to dissolve **9** in DMSO-*d*_6_ and record the spectrum, bubble ethylene directly into the NMR tube and record the spectra again. Free ethylene resonance was observed at 5.14 ppm, in DMSO-*d*_6_ and it was concluded that no reaction took place. The addition of a proton source was attempted to see the reaction chemistry. Trifluoroacetic acid (TFA) and triflic acid (TFMS) were selected as proton sources, since TFA is a weak acid and TFMS a strong acid, and neither have interfering proton resonances. Portions ranging from ~10 to 20-fold excess of TFA or TFMS were added to nine and ethylene mixtures, and the spectra were recorded again. It was assumed that the palladium complexes would be fairly tolerant of an acidic environment, due to the fact that Pd(II) can chelate peptide amide bonding at low pH values [17].

**Scheme 3:**
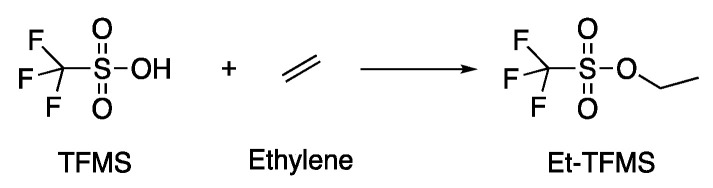
Oxidation of ethylene to form acetaldehyde catalyzed by pd(II).

Increasing volumes of TFA to a solution of **9** and ethylene resulted in the demetallation of the ligand, where above a ~15-fold excess, the spectra revealed complete demetallation and protonation of the N functional groups in the presence of acidic protons. In parallel, increased amounts of TFA resulted in the oxidation of ethylene to form acetaldehyde (See Scheme 3). The formation of acetaldehyde increased proportionally with the quantity of TFA and appeared catalyzed by Pd(II) after dissociation from the ligand. With a reversed order of addition, first TFA then ethylene, acetaldehyde only formed once both TFA and ethylene were present. A blank spectrum of TFA and ethylene in DMSO confirmed that this was not a spontaneous reaction. 

**Scheme 4:**
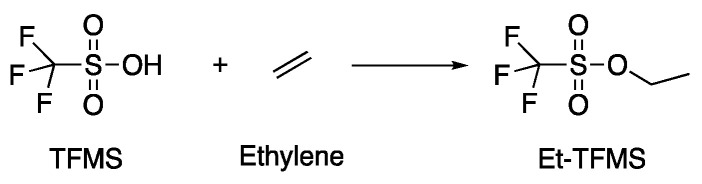
Spontaneous reaction between trifluoromethanesulfonate and ethylene to form ethyl trifluoromethanesulfonate.

The triflic acid (tfms) was substituted for tfa to determine if a different proton source produces the same product; dissimilar to tfa, tfms is non-coordinating. The addition of excess TFMS also resulted in the demetallation of the ligand, and again the oxidation of ethylene was observed by the formation of acetaldehyde signals. This reaction also formed a second product that was likely ethyl trifluoromethanesulfonate (Scheme 4), which is known to form from triflic acid in the presence of ethylene [50,51]. 

The ^13^C-NMR spectra for this experiment revealed that the addition of 20–30 μL TFA did not result in any significant changes to the signals of **9**; although this amounted to ~10 to 15-fold excess TFA, the signature quadruplets for TFA at ~158 and ~115 ppm were not present in the spectrum. This could be related to the volatility of this reagent and the length of ^13^C experiments. When more TFA (40 μL) or TFMS (40 μL) are added, the ^13^C signals were affected significantly. This supports the findings that demetallation occurred. The confirmation of Et-TFMS formation was seen in the corresponding ^13^C signals at 15.67 and 73.08, respectively. The formation of an aldehyde signal at 201.25 ppm and the appearance of a peak at 31.10 ppm supports the assertion that acetaldehyde was formed.

Complex **10**, with the more labile six-membered ring, was also reacted with TFA and ethylene. It was expected in a direct comparison with **9**; complex **10** would show a higher reactivity. Upon TFA addition to 10, the ^1^H-NMR changed dramatically. Again, partial demetallation and amide protonation occurred. However, a large shift in the Asp moiety, still coupled with the Asp NH_2_, indicated that the amine was still coordinated. After the addition of ethylene, the formation of acetaldehyde was observed in about ~10% quantitatively relative to the complex. The ^13^C-NMR spectrum for this experiment was not informative due to the lability of the complex. The complexes **9**–**11** were; therefore, not amenable to ethylene coordination despite its stronger ligand properties compared to the carbonyl ester.

#### 2.5.3. Derivatives of CO_2_ and CO

Carbonylation reactions are typically performed using CO in conjunction with various monomers, including olefins [52,53,54] and alcohols [55] under high pressure to yield various carboxylic acid derivatives. In order to avoid the use of carbon monoxide as C_1_-feedstock, CO surrogates may be used. Chief among these CO alternative are formic acid derivatives [56]. 

In order to examine whether these complexes were candidates for carbonyl coordination, the activation of methyl formate was investigated. A stock solution of methyl formate in DMF was added to solutions of 9–10 in a 1.1:1 ratio, with the intent of monitoring the electronic spectra. However, upon mixing, the formation and precipitation of Pd black occurred immediately. Coordination of this formate was, therefore, not successful, although these results did not accurately predict its potential performance in carbonylation reactions since carbonylation reactions require a protic solvent or a proton source as well as a base [56]. Acidic conditions such as employed in the Wacker process led to the reductive elimination and reduction to Pd(0).

## 3. Discussion

The synthesis and characterization of tetradentate tripeptide complexes **9**–**11** with C-terminal carbonyl ester coordination were reported. The complexes were designed to form hemilabile ligand chelates that could open and close depending on the surrounding system. However, this coordination mode formed weaker sigma bonds which made it inherently more difficult to form in the presence of a more favorable ligands. The development of a non-aqueous synthesis strategy, using a non-nucleophilic base was instrumental in the formation of neutral tetradentate palladium complexes with κ^4^[NH_2_,N,N,=O] coordination. Charged ester carbonyl palladium complexes are reported in the literature, [39] but neutral complexes appear to be much rarer. The use of this synthetic strategy offers the possibility to facilitate the coordination of ester carbonyl oxygen to other metal centers. 

Pd(II) complexes with tridentate tripeptides **4**–**6** were also isolated. These complexes were anionic and had a chloride ligand. Ligand exchange reactions of **4** successfully displaced the chloride ligand with lutidine (**8**) and pyridine (**7**). However, lutidine was a weaker nucleophile and was sterically more hindered than pyridine, resulting in were incorporation. Lutidine exhibited partial thermal lability resulting in ~30% dissociation upon heating to form complex **9**.

The development of this class of complexes was meant to achieve maximum organosolubility, to carry out reactions in non-coordinating solvents. Despite **9** and **10** having multiple functional groups, including the methyl and *t-*butyl esters, and an indole present **11**, these complexes were relatively insoluble. Consequently, characterization and reaction studies were carried out in coordinating solvents. 

Complexes **9**–**11** Ware able to catalytically transfer sulfur atoms, where **10** outperformed **9** and **11** in terms of TOF before deactivation. The catalytic studies of synthesized Pd complexes with cyclohexene sulfide showed that **10**, with its more labile six-membered chelate, was more reactive. The study confirmed that the Pd complexes are capable of catalyzing the reaction of cyclohexene sulfide to cyclohexene. The complexes also appeared to form polythioethers but were not sufficiently active to be considered more than modest catalysts for sulfur transfer compared to a recent report of TOF of 698 h^−1^ for a similar reaction [57]. 

In the presence of Pd(II), ethylene was oxidized to form the acetaldehyde in the well-known Wacker Process (WP) [58]. The reactivity studies of **9** and **10** with ethylene showed the activation of the ethylene group only with the addition of a proton source. After the addition of ~10-fold excess acid to the complex and olefin, demetallation was observed. The degree of demetallation increased with the amount of acid, and the amount of acetaldehyde formed also increased. It was unclear if the formation of acetaldehyde was limited by the number of acidic protons or the oxidizing agent, namely, trace amounts of water. Complexes **9** and **10** both exhibited a modest ability to convert ethylene to acetaldehyde, but it was unclear if this was due solely to ligand demetallation. Tripeptide palladium complexes synthesized in this work appeared susceptible to reduction to Pd(0) in the presence of CO and CO surrogates. 

An interesting outcome was that demetallation was a recurring theme and olefin coordination did not take place. The ligand donors were sigma donors only and may have led to a very electron-rich metal center for lower oxidation states. The carbonyl ester coordination appeared stronger than presumed as was borne out in comparison of the reported hemilabile Pd–OMe bond distance of 2.178(3) Å [49] and the calculated distance of 2.109 Å. Considering the carbonyl oxygen donor may serve as a π acceptor and an electron, a rich metal center may relieve its electron density through this bond. 

## 4. Materials and Methods

Reagents used were purchased from Sigma-Aldrich and used without further purification unless otherwise stated. Solvents were purchased from Sigma-Aldrich and were distilled under nitrogen and dried using standard methods [59]. Ligands **1**–**3** were prepared as outlined in previous work [31]. [Pd(CH_3_CN)_2_Cl_2_] was prepared according to a published procedure [60]. 

Infrared spectra were recorded on a Nicolet Avatar 360 FT-IR (E.S.P.) spectrophotometer (Thermo Fisher Scientific, Waltham, MA, USA) using κBr pellets. The ^1^H, COSY, and ^13^C nuclear magnetic resonance spectra were recorded at ambient temperature on a Bruker AVANCE 400 MHz spectrometer (Bruker, Billerica, MA, USA) at 400 and 101 MHz, respectively. Solvents used were D_2_O, DMSO-*d*_6_, and CDCl_3_. Electronic spectra were obtained using either Varian Cary 100 Bio spectrophotometer (Agilent, Santa Clara, CA, USA) or PerkinElmer Lambda 25 UV/Vis spectrophotometer (Thermo Fisher Scientific, Waltham, MA, USA). Mass spectra were recorded on a micrOTOF-Q spectrometer (Bruker, Billerica, MA, USA), equipped with E-spray atmospheric pressure ionization chamber (ESI). Elemental analyses were obtained from Midwest Microlab, Indianapolis, IN, USA.

### 4.1. Synthesis

#### 4.1.1. K[Pd{α-Asp(O*^t^*Bu)AlaGly(OMe)}Cl] (**4**)

K_2_PdCl_4_ (0.040 g, 0.136 mmol) and 1 (0.050 g, 0.136 mmol) were dissolved in H_2_O (5 mL) and KOH was added until pH = 6.5. Solution was stirred overnight under N_2_. The H_2_O was removed in vacuo and the product was isolated by dissolving in EtOH and filtering (0.055 g, 98.2%): ^1^H-NMR (400 MHz, DMSO-*d*_6_) δ 4.20 (dd, 1H, NH_Asp_), 3.84 (d, 1H, NH_Asp_), 3.69–3.57 (m, 2H, α-H_Gly_), 3.56–3.51 (m, 1H, α-H_Ala_), 3.50 (s, 3H, -OCH_3 Gly_), 3.43–3.36 (m, 1H, α-H_Asp_), 2.48–2 (m, 1H, β-H_Asp_), 2.35–2.29 (m, 1H, β-H_Asp_), 1.40 (s, 9H, -O(CH_3_)_3 Asp_), 1.15 (d, 3H, β-H_Ala_). IR (KBr) cm^−1^: 3315, 3234 (b, ν(N-H)), 1723 (s, ν(C=O)), 1585 (s, ν(C=O)), 1570 (s, ν(C-N)), 1254 (s, ν(C-O)), 1210 (s, ν(C-O)), 1148 (s, ν(C-O)). UV–VIS (H_2_O): ε_x_ MS (ESI/Negative); MW: (C_14_H_23_N_3_O_6_PdCl) = 471.2230, *m*/*z* found(calc) = 472.0311(472.0313) [M^−^].

#### 4.1.2. (Et_3_N)[Pd{α-Asp(O*^t^*Bu)AlaGly(OMe)}Cl] (**5**)

Pd(CH_3_CN)_2_Cl_2_ (0.076 g, 0.297 mmol) and **1** (0.097 g, 0.297 mmol) were dissolved in dry THF (~20 mL) and triethylamine (0.08 mL, 0.585 mmol) was added. The reaction was stirred overnight under Ar. Solution was filtered through celite and removed in vacuo. Seven-fold excess Et_3_NCl salt (crude yield 0.097 g): ^1^H-NMR (400 MHz, DMSO-*d*_6_) δ 4.21 (dd, 1H, NH_Asp_), 3.85 (t, 1H, NH_Asp_), 3.6–3.56 (m, 2H, α-H_Gly_), 3.56–3.51 (m, 1H, α-H_Ala_), 3.50 (s, 3H, -OCH_3 Gly_), 3.40 (d, 1H, α-H_Asp_), 3.08 (q, 14H, -CH_2 TEA_), 2.44 (d, 1H, β-H_Asp_), 2.33 (dd, 1H, β-H_Asp_), 1.40 (s, 9H, O(CH_3_)_3 Asp_), 1.23–1.13 (m, 24H, O(CH_3_)_3 Asp_, -CH_3 TEA_). IR (KBr) cm^−1^: 3313, 3215 (b, ν(N-H)), 1750 (s, ν(C=O)), 1724 (s, ν(C=O)), 1601 (s, ν(C=O)), 1583 (s, ν(C-N)), 1250 (s, ν(C-O)), 1210 (s, ν(C-O)), 1148 (s, ν(C-O)).

#### 4.1.3. (PPh_4_)[Pd{α-Asp(O^t^Bu)AlaGly(OMe)}Cl] (**6**)

Excess PPh_4_Cl (0.128 g, 0.293 mmol) and crude **5** (0.097 g) were dissolved in dry THF. The resulting precipitate was filtered: ^1^H-NMR (400 MHz, Chloroform-*d*) δ 7.98–7.91 (m, 4H, PPh*_para_*), 7.81 (td, 8H, PPh*_meta_*), 7.69–7.60 (m, 8H, PPh*_ortho_*), 4.01–3.85 (q, 2H, α-H_Gly_), 3.96 (m, 1H, α-H_Ala_), 3.64 (d, 1H, α-H_Asp_), 3.58 (s, 3H, OCH_3 Gly_), 2.82 (d, 1H, β-H_Asp_), 2.50 (dd, 1H, β-H_Asp_), 1.42 (s, 9H, -O(CH_3_)_3 Asp_), 1.35 (d, 3H, β-H_Ala_). IR (KBr) cm^−1^: 3314, 3215 (b, ν(N-H)), 1750 (s, ν(C=O)), 1724 (s, ν(C=O)), 1601 (s, ν(C=O)), 1583 (s, ν(C-N)), 1250 (s, ν(C-O)), 1194 (s, ν(C-O)), 1138 (s, ν(C-O)).

#### 4.1.4. Pd{α-Asp(O*^t^*Bu)AlaGly(OMe)}(Lu) (**7**)

**1** (0.097 g, 0.293 mmol) and 2,6-lutidine (0.064 mL, 0.585 mmol) were dissolved in THF (20 mL). Pd(OAc)_2_ (0.066 g, 0.293 mmol) was added, and the reaction stirred under Ar overnight. The solution was filtered, the volume reduced in vacuo and precipitated out and washed with Et_2_O (0.113 g 88.3%): ^1^H-NMR (400 MHz, DMSO-*d*_6_) δ 7.78 (t, 1H, Lu*_para_*), 7.33 (dd, 2H, Lu*_meta_*), 4.76 (dd, 1H, NH_Asp_), 4.15 (t, 1H, NH_Asp_), 3.71–3.60 (m, 1H, α-H_Ala_), 3.54 (q, 1H, α-H_Asp_), 3.34 (s, 3H, -OCH_3 Gly_), 3.18 (s, 6H, Lu_CH3_), 3.14 (d, 1H, α-H_Gly_), 2.91 (d, 1H, α-H_Gly_), 2.57 (dd, 1H, β-H_Asp_), 2.45–2.34 (m, 1H, β-H_Asp_), 1.41 (s, 9H, -O(CH_3_)_3 Asp_), 1.23(m, 3H, β-H_Ala_); ^13^C NMR (101 MHz, DMSO) δ 185.45 (C=O_Asp_), 175.90 (C=O_Ala_), 171.70 (C=O_Gly_), 169.93 (γ-C=O_Asp_), 160.12 (Lu*_ortho_*), 159.69 (Lu*_ortho_*), 139.08 (Lu*_para_*), 122.72 (Lu*_meta_*), 122.60 (Lu*_meta_*), 80.14 (OC(CH_3_)_3 Asp_), 57.13 (OCH_3 Gly_), 56.50 (α-C_Asp_), 50.79 (α-C_Ala_), 45.84 (α-C_Gly_), 39.73 (β-C_Asp_), 27.79 ((OC(CH_3_)_3 Asp_), 25.86 (Lu_CH3_), 25.77 (Lu_CH3_), 19.78 (β-C_Ala_). IR (KBr) cm^−1^: 3303, 3223 (b, ν(N-H)), 1750 (s, ν(C=O)),1732 (s, ν(C=O)), 1604 (s, ν(C=O)), 1582 (s, ν(C-N)), 1250 (s, ν(C-O)), 1200 (s, ν(C-O)), 1147 (s, ν(C-O)). MS (ESI/Positive) MW: (C_21_H_32_N_4_O_6_Pd) = 542.9290, *m*/*z* found(calc) = 565.1254(565.1257) [M + Na^+^].

#### 4.1.5. Pd{α-Asp(O*^t^*Bu)AlaGly(OMe)} (**9**)

**1** (0.331 g, 1.000 mmol) and Proton Sponge (0.536 g, 2.500 mmol) were dissolved in THF (20 mL). Pd(OAc)_2_ (0.224 g, 1.000 mmol) was added, and the reaction stirred under Ar for 5 h. The solution was concentrated in vacuo and precipitated out with several additions of EtOAc and toluene. Product was recrystallize as a purple solid from acetonitrile and EtOAc (0.295 g, 67.7%) ^1^H-NMR (400 MHz, DMSO-*d*_6_) δ 5.04 (t, 1H, NH_Asp_), 4.55 (t, 1H, NH_Asp_), 3.95–3.79 (m, 2H, α-H_Gly_), 3.68 (q, 1H, α-H_Ala_), 3.56 (s, 1H, α-H_Asp_), 3.55 (s, 3H, -OCH_3 Gly_), 2.61 (dd, 1H, β-H_Asp_), 2.48–2.42 (m, 1H, β-H_Asp_), 1.42 (s, 9H, -O(CH_3_)_3 Asp_), 1.20 (d, 3H, β-H_Ala_); ^13^C NMR (101 MHz, DMSO) δ 177.03(C=O_Asp_), 172.89(C=O_Ala_), 169.79(γ-C=O_Asp_), 99.49(C=O_Gly_), 80.47(-OC(CH_3_)_3 Asp_), 56.59(OCH_3 Gly_), 55.80(α-C_Asp_), 50.93(α-C_Ala_), 46.79(α-C_Gly_), 39.94(β-C_Asp_), 27.79(-OC(CH_3_)_3 Asp_), 19.80(β-C_Ala_). IR (KBr) cm^−1^: 3313, 3245 (b, ν(N-H)), 1731 (s, ν(C=O)), 1588 (s, ν(C=O)), 1541 (s, ν(C-N)), 1253 (s, ν(C-O)), 1206 (s, ν(C-O)), 1145 (s, ν(C-O)). UV–Vis (DMSO): 𝜀_335_ = 1520 L/mol⋅cm MS (ESI/Positive) MW: (C_14_H_25_N_3_O_6_Pd) = 435.7730, *m*/*z* found(calc) = 458.0515(458.0520) [M + Na^+^]. CHN (C_14_H_23_N_3_O_6_Pd) found(calc)%: C: 38.36(38.59), H: 5.25(5.32), N: 9.64(9.64).

Route 2: α-D(O*^t^*Bu)AG(OMe) (0.120 g, 0.362 mmol) dissolved in 10 mL H_2_O was added to an aqueous solution of K_2_PdCl_4_ (0.146 g, 0.362 mmol) in 20 mL H_2_O. The pH was increased to 10 with KOH and stir for 0.5 h. The water was removed, and the product was isolated by dissolving in acetonitrile and filtering. Presence of **8** was confirmed spectroscopically, but purification was unsuccessful.

#### 4.1.6. Pd{β-Asp(O*^t^*Bu)AlaGly(OMe)} (**10**)

Ligand **2** (0.331 g, 1.000 mmol) and Proton Sponge (0.536 g, 2.500 mmol) were dissolved in THF (20 mL). Pd(OAc)_2_ (0.224 g, 1.000 mmol) was added, and the reaction stirred under Ar for 5 h. The solution was concentrated in vacuo and precipitated out with several additions of EtOAc and toluene. Product was recrystallized as a red–orange solid from acetonitrile and EtOAc (0.287 g, 65.8%) ^1^H-NMR (400 MHz, DMSO-*d*_6_) δ 4.62 (m, 1H, NH_Asp_), 4.48 (m, 1H, NH_Asp_), 3.95 (m, 1H, α-H_Ala_), 3.72–3.52 (m, 2H, α-H_Gly_), 3.56 (s, 3H, -OCH_3 Gly_), 3.18 (s, 1H, α-H_Asp_), 2.44–2.22 (m, 2H, β-H_Asp_), 1.44 (s, 9H, -O(CH_3_)_3 Asp_), 1.08 (d, 3H, β-H_Ala_); ^13^C NMR (101 MHz, DMSO) δ 172.88(C=O_Asp_), 171.14(C=O_Ala_), 170.16 (γ-C=O_Asp_), 99.49(C=O_Gly_), 82.32 (OC(CH_3_)_3 Asp_), 51.26 (OCH_3 Gly_), 54.70 (α-C_Asp_), 59.14 (α-C_Ala_), 46.20 (α-C_Gly_), 41.09 (β-C_Asp_), 28.13 (OC(CH_3_)_3 Asp_), 21.07 (β-C_Ala_). IR (KBr) cm^−1^: 3313, 3245 (b, ν(N-H)), 1731 (s, ν(C=O)), 1588 (s, ν(C=O)), 1541 (s, ν(C-N)), 1253 (s, ν(C-O)), 1206 (s, ν(C-O)), 1145 (s, ν(C-O)). UV–Vis (DMSO): ε_333_ = 1260 L/mol⋅cm MS (ESI/Positive) MW: (C_14_H_25_N_3_O_6_Pd) = 435.7730, *m*/*z* found(calc) = 458.0515(458.0520) [M + Na^+^]. CHN (C_14_H_23_N_3_O_6_Pd) C: 38.28(38.59), H: 5.24(5.32), N: 9.69(9.64).

#### 4.1.7. Pd{TrpAlaGly(OMe)} (**11**)

Ligand **3** (0.143 g, 0.413 mmol) and Proton Sponge (0.221 g, 1.032 mmol) were dissolved in THF (20 mL). Pd(OAc)_2_ (0.093 g, 0.413 mmol) was added, and the reaction stirred under Ar for 5 h. The solution was concentrated in vacuo and precipitated out with Et_2_O. The orange solid was collected and washed with portions of EtOAc and Et_2_O. Hot CH_3_CN was used to redissolve the product, product was filtered and concentrated in vacuo. (0.169 g, 90.0%); ^1^H-NMR (400 MHz, DMSO-*d*_6_) δ 10.90 (d, 1H, NH_indole_), 7.55 (d, 1H, H_indole-C7_), 7.37 (d, 2H, H_indole-C4_), 7.27 (d, 2H, H_indole-C2_), 7.09 (ddd, 1H, H_indole-C5_), 6.99 (ddd, 1H, H_indole-C6_), 4.96 (t, 1H, NH_Trp_), 4.24 (t, 1H, NH_Trp_), 3.96 (d, 1H, α-H_Gly_), 3.83 (d, 1H, α-H_Gly_), 3.82–3.75 (m, 2H, α-H_Ala_), 3.55 (s, 4H, ROCH_3 Gly_), 3.40 (dt, 2H, α-H_Trp_), 3.28 (dd, 1H, β-H_Trp_), 2.87 (dd, 2H, β-H_Trp_), 1.24 (d, 3H, β-H_Ala_); ^13^C-NMR (101 MHz, DMSO) δ 178.24(C=O_Trp_), 172.93(C=O_Ala_), 136.37(C3_indole_), 127.11(C8_indole_), 123.99(C2_indole_), 121.10(C5_indole_), 118.44(C6_indole_), 118.24(C7_indole_), 111.41(C4_indole_), 109.47(C1_indole_), 67.00, 59.58(α-C_Gly_), 56.50(OCH_3 Gly_), 50.93(α-C_Ala_), 46.86(α-C_Gly_), 29.93(β-C_Trp_), 19.92(β-C_Ala_). IR (KBr) cm^−1^: 3370, 3286, 3235(b, ν(N-H)), 1741(s, ν(C=O)), 1569(sh)(s, ν(C=O)), 1543(ν(C-N)), 1333, 1070, 1010(ν(C-N)), 1105(sh), 1025, 981(ν(C-N)), 1207 (ν(C-O)), 1178 (s, ν(C-O)). UV–Vis (DMSO): ε_317_ = 3239, ε_344_ = 2188 L/mol·cm MS (ESI/Positive) MW: (C_17_H_20_N_4_O_4_Pd) = 450.7910, *m*/*z* found(calc) = 437.0417(437.0419) [M + Na^+^]. CHN (C_17_H_20_N_4_O_4_Pd) found(calc)%: C: 44.51(43.55), H: 4.73(4.73), N: 11.47(11.95).

### 4.2. Reaction Studies

#### 4.2.1. Sulfur Reagents

Samples of **9**–**11** were prepared aerobically by adding cyclohexene sulfide (0.3833 mmol) to DMSO-*d*_6_ (0.6 mL) in screw-cap NMR tub. Initial ^1^H-NMR spectrum were recorded at ambient temperature and pressure to obtain a blank spectrum. Complexes **9**–**11** (0.0115 mmol) were added to NMR tube and ^1^H-NMR spectrum for recorded instantly. The progress of the reaction was monitored over the course of hours or days to determine conversion of thiirane to alkene. Maintaining conditions but omitting the catalyst showed no identifiable alkene formation over commensurate time periods as the catalytic runs.

#### 4.2.2. Ethylene and Acids

NMR tube was charged with complex **9** or **10** (10.1 mg, 0.023 mmol) and dissolved in 650 μL of DMSO-d6, the NMR recorded, then ethylene was bubbled in for 1 min, and the NMR recorded again (Figure S10). TFA (~20 μL, 0.128 mmol; ~30 μL, 0.192 mmol; ~40 μL (0.256 mmol) or TFMS (~40 μL, 0.443 mmol) was added to the NMR tube and the spectrum recorded again. Complex **8** (9.7 mg, 0.022 mmol) was dissolved in 650 μL of DMSO-d6, then TFA (~20 μL, 0.128 mmol) was added and the spectrum recorded; ethylene was bubbled in and the NMR recorded again.

### 4.3. Quantum Chemical Calculations

Calculations were performed with version 4.2.1 of the ORCA program [61,62]. The density functional theory based protocol consisted of the PBE0-D3BJ functional [63,64,65] (including the D3 dispersion correction by Grimme and coworkers [66,67]) and the triple-zeta basis set def2-TZVP [68]. The RIJCOSX [69,70] approximation was used to calculate Coulomb and Exchange integrals, using the def2/J auxiliary basis set by Weigend et al. [71] and the GridX5 (ORCA keyword) grid was used. Tighter grids for the exchange-correlation terms were also used (Grid5, FinalGrid6 keywords in ORCA). The CPCM solvation model [72,73,74] using a Gaussian point charge scheme and a scaled vdW surface was used to incorporate solvation effects. Vibrational frequencies were calculated analytically, as implemented in ORCA [75,76]. The ^13^C-NMR shieldings were calculated using the same level of theory, except the pcSseg-2 basis sets [77] were utilized on carbon and hydrogen atoms. The calculated shieldings were converted into chemical shifts by calculating the shielding difference with respect to tetramethylsilane at the same level of theory. Chemical shifts were shifted by −15 ppm due to a systematic overestimation.

## Figures and Tables

**Table 1:** Calculated bond angles (°) and lengths (Å) for complexes **4**, **8**–**11**. X = Cl (**4**), Lu (**8**), or -OMe (**9**, **10**, **11**).

Bond Angle	4	8	9	10	11
NH_2_-Pd-N_ala_	82.84	82.71	82.97	95.01	82.61
N_ala_-Pd-N_gly_	81.92	82.11	83.58	83.17	83.68
N_gly_-Pd-X	99.54	98.35	80.47	80.99	80.32
X-Pd-NH_2_	95.70	96.88	112.90	100.54	113.38
**Bond Length**					
NH_2_-Pd	2.061	2.060	2.077	2.064	2.073
N_ala_-Pd	1.940	1.942	1.916	1.964	1.911
N_gly_-Pd	1.996	1.996	1.937	1.936	1.938
X-Pd	2.355	2.070	2.142	2.109	2.142

**Table 2:** Summary of ^1^H NMR data: **4** = K[Pd(**1**)Cl], **7** = Pd(**1**)Py, **8** = Pd(**1**)Lu, **9** = Pd(**1**), **10** = Pd(**2**), **11** = Pd(**3**).

Chemical Shift (ppm)	4	7	8	8 + Δ *	9	10	11
Py*_Ortho_*	-	8.73	-	-	-	-	-
Py/Lu*_Meta_*	-	7.57	7.33	7.02	-	-	-
Py/Lu*_Para_*	-	7.99	7.78	7.54	-	-	-
N-H_2_	4.20 3.86	4.96 4.38	4.76 4.15	5.03 4.54	5.04 4.55	4.63 4.48	4.96 4.24
α-C_Ala_	3.54	3.66	3.66	3.68	3.68	3.89	3.78
α-C_Gly_	3.64	3.52	3.14 2.91	3.86	3.87	3.62	3.96
α-C_Trp/Asp_	3.40	3.44	3.54	3.53	3.52	2.33	3.40
OCH_3 Gly_	3.50	3.34	3.34	3.55	3.55	3.55	3.55
Lu CH_3_	-	-	3.18	2.41	-	-	-
β-C_Trp/Asp_	2.45 2.35	2.61 2.40	2.57 2.41	2.63 2.46	2.61 2.45	3.18	3.28 2.87
OC(CH_3_)_3 Asp_	1.40	1.40	1.41	1.42	1.42	1.44	-
β-C_Ala_	1.15	1.23	1.23	1.20	1.20	1.08	1.24

* Represents the minor species.

**Table 3:** Assigned ^13^C-NMR resonances for complexes **4**, **8**–**11**.

Chemical Shift (ppm)	4	8 *	9	10	11
C=O_Trp/Asp_	170.14	169.93	169.79	170.75	172.93
C=O_Ala_	185.17	185.45	177.03	176.26	178.24
γ-C=O_Asp_	175.91	175.9	172.89	172.58	-
C=O_Gly_	172.34	171.71	186.47	n.d.	186.55
OC(CH_3_)_3 Asp_	79.93	80.14	80.47	82.32	-
OCH_3 Gly_	50.52	50.79	50.96	50.92	50.93
α-C_Trp/Asp_	56.55	56.50	55.81	40.16	59.58
α-C_Ala_	57.05	57.13	56.60	58.87	56.50
α-C_Gly_	46.49	45.84	46.79	44.73	46.86
β-C_Trp/Asp_	39.86	39.73	39.94	54.7	29.93
OC(*C*H_3_)_3 Asp_	27.79	27.79	27.79	27.58	-
β-C_Ala_	19.43	19.78	19.80	20.64	19.92

* Trp side chain and lutidine resonances found in experimental section. n.d.—not observed.

**Table 4:** Observed and assignment of ^13^C NMR resonances for **4**, **8**–**11** and corresponding calculated values.

Chemical Shift (ppm)	4 (obs)	4 (*calc*)	8 (obs)	8 (*calc*)	9 (obs)	9 (*calc*)	10 (obs)	10 (*calc*)	11 (obs)	11 (*calc*)
C=O_Trp/Asp_	170.14	170.82	169.93	171.07	169.79	171.39	170.75	165.3	172.93	169.9
C=O_Ala_	185.17	181.86	185.45	182.5	177.03	177.63	176.26	176.5	178.24	177.11
γ-C=O_Asp_	175.91	171.72	175.9	174.71	172.89	172.31	172.58	172.4	-	-
C=O_Gly_	172.34	171.34	171.71	173.3	186.47	186.23	n.d.	187.8	186.55	185.82

n.d.—not observed.

**Table 5:** Summary of infrared data for **1**–**4**, **8–11**.

Frequency (cm^−1^)	1	2	3	4	8	9	10	11
ν(NH_2_) ν(NH)	3370 3314	3375 * 3304	3370 * 3297	3234 3138	3223 3100	3245 3129	3239 3121	3286 3235
ν(C=O)								
O*^t^*Bu	1725	1743	-	1723	1732	1729	1732	-
OMe	1759 *	1757 *	1749	1748 *	1750 *	1541	1551	1554
Amide I	1670	1659	1655	1585 1570	1604 1582	1608 1584	1590 1567	1598 1569
ν(C-O)								
O*^t^*Bu	1250	1254	-	1254	1250	1253	1258	-
OMe	1213	1212	1213	1210	1210	1205	1207	1207
ν(O-C)								
O*^t^*Bu	1155	1155	-	1148	1147	1145	1155	-
ν(M-N)	1250	1254	-	573 548	568 548	573 548	573 533	568 534

* Denotes shoulder absorption.

**Table 6:** Comparative frequencies (cm^−1^) for ν(C=O) vibrational modes observed versus DFT-calculated for **4**, **8**–**11**. The Δ values show the deviation between observed and calculated DFT values.

Complex	ν(C=O) O*^t^*Bu	ν(C=O) OMe	ν(C=O) Amide I	ν(C=O) Amide I
**4** Obs	1723	1748	1585	1570
DFT	1767	1782	1659	1639
Δ	44	34	74	69
**8** Obs	1732	1750	1604	1582
DFT	1749	1790	1664	1642
Δ	17	40	60	60
**9** Obs	1729	1541	1608	1584
DFT	1757	1677	1666	1652
Δ	28	136	58	68
**10** Obs	1732	1551	1590	1567
DFT	1784	1674	1653	1653
Δ	52	123	63	86
**11** Obs	-	1554	1598	1569
DFT	-	1652	1677	1662
Δ	-	98	79	93

## Data Availability

Not applicable.

## Supplementary Materials

The following are available online. Figures S1–S6: The ^1^H-NMR, ^13^C-NMR and MS for **4**, **7**, **8**–**11**, Tables S1 and S2: The ^1^H and ^13^C-NMR data for **1**–**3**, Figure S7: stacked IR spectra for **1**, **4**, **8**, and **9′** Figure S8: the ^1^H-NMR spectra for **10** in DMSO-*d*_6_: sulfur transfer reactions, Figure S9: example of reaction progress for SAT by **10** in DMSO-*d*_6_, Figure S10: stacked ^1^H-NMR spectra of reactions of **9** (1) with ethylene and acids. DFT generated cartesian coordinates for complexes **4**, **8**–**11** in Ångstrom.

## Sample Availability

Samples of the compounds are not available from the authors.
