# Supplementary material for: Synthesis, Characterization, and Reaction Studies of Pd(II) Tripeptide Complexes
Source: Molecules. 2021 Aug 26;26(17):5169. doi: 10.3390/molecules26175169 (PMC8433849; doi:10.3390/molecules26175169)
Supplement: Supplementary file 1 [file molecules-26-05169-s001.zip › molecules-1336862-supplementary-NEW.pdf]

Synthesis, Characterization, and Reaction Studies of Pd(II) Tripeptide Complexes

Lindsey J. Monger [1], Dmitrii Razinkov [1], Ragnar Bjornsson [2] and Sigridur G. Suman [1,*]

[1] Science Institute, University of Iceland, Dunhagi 3, 107 Reykjavik, Iceland
[2] Max Planck Institute Chemical Energy Conversion, Mülheim an der Ruhr, Germany
* Correspondence: sgsuman@hi.is.

Supplementary Information.

Figure S1: ^{1}H NMR spectra for **4**. Figure S1b: MS scan for **4**.
Figure S2a: ^{1}H NMR spectra for **7**.
Figure S3a: ^{1}H NMR spectra for **8**. Figure S3b: MS scan for **8**.
Figure S4a: ^{1}H NMR spectra for **9**. Figure S4b: ^{13}C NMR spectra for **9**. Figure S4c: MS scan for **9**. Figure S5a: ^{1}H NMR spectra for **10** at 330K. Figure S5b: ^{13}C NMR spectra for **10** at 330K. Figure S5c: MS scan for **10**.
Figure S6a: ^{1}H NMR spectra for **11**. Figure S6b: ^{13}C NMR spectra for **11**. Figure S6c: MS scan for **11**.
Table S1: ^{1}H NMR spectra for 1-3.
Table S2: ^{13}C NMR spectra for 1-3.
Table S3: Select functional group IR vibrations for 1-3.
Figure S7: Stacked IR spectra for **1**, **4**, **8**, and **9**.
Figure S8: ^{1}H NMR spectra for **10** in DMSO-d_6: 1) before and 3) after catalytic sulfur transfer reactions. Compared to 2) free ligand 2. NMR suggests complex is demetallized.
Figure S9: Example of reaction progress for cyclohexene sulfide SAT by **10** in dmso-d_6.
Figure S10: Stacked ^{1}H NMR spectra of **9** (1) with addition of: (2) 30 µL TFA, (3) Ethylene, (4) 20 µL TFA and Ethylene, (5) 30 µL TFA and Ethylene, (6) 40 µL TFA and Ethylene, (7) 40 µL TFMS and Ethylene, (8) Ligand 1, (9) Blank with Ethylene and TFA.
DFT generated cartesian coordinates for complexes **4**,**8**-**11** in Ångstrom.

Figure S1a

^{1}H NMR spectra for **4**.

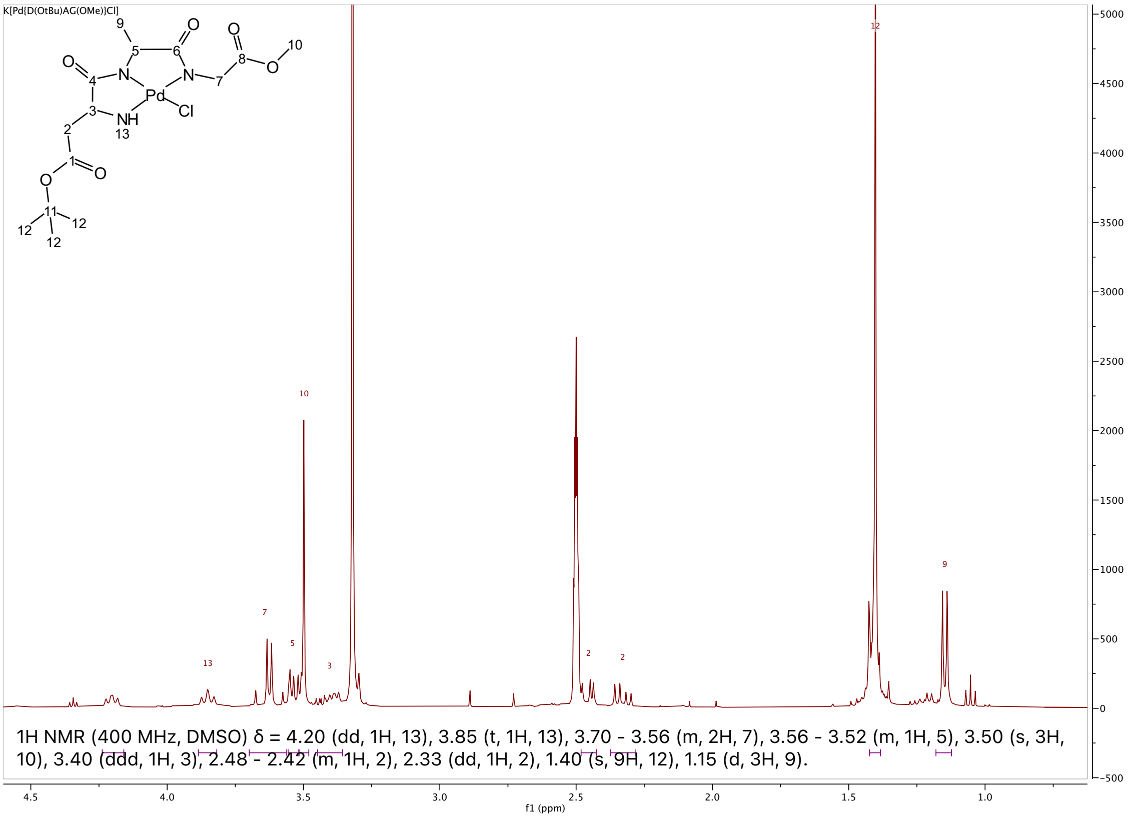

Figure S1b

MS scan for **4**.

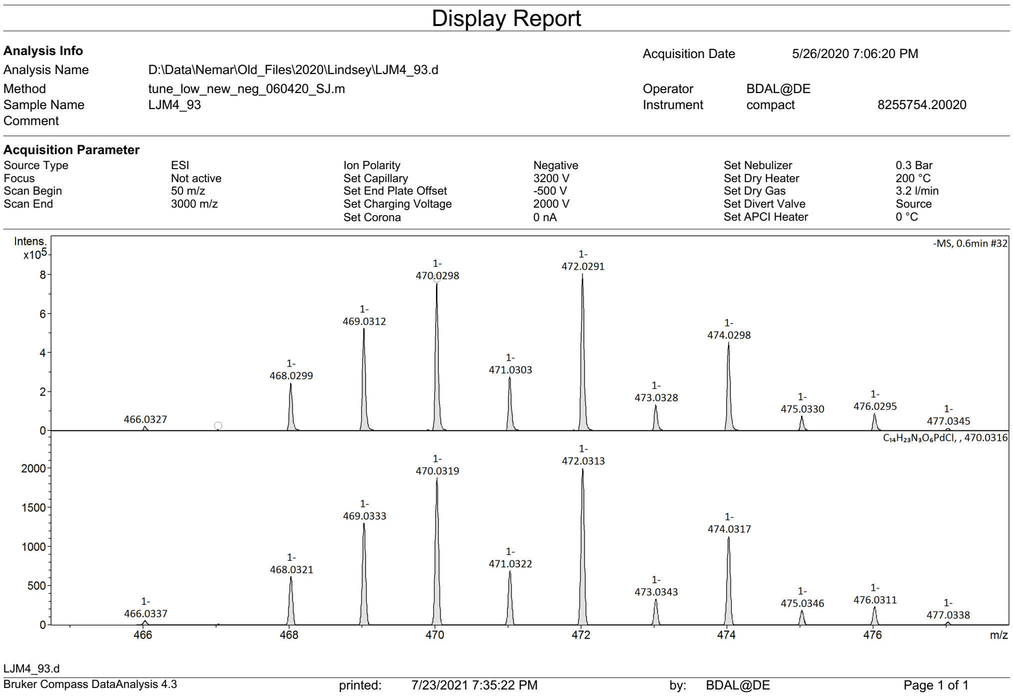

Figure S2a

^{1}H NMR spectra for **7**.

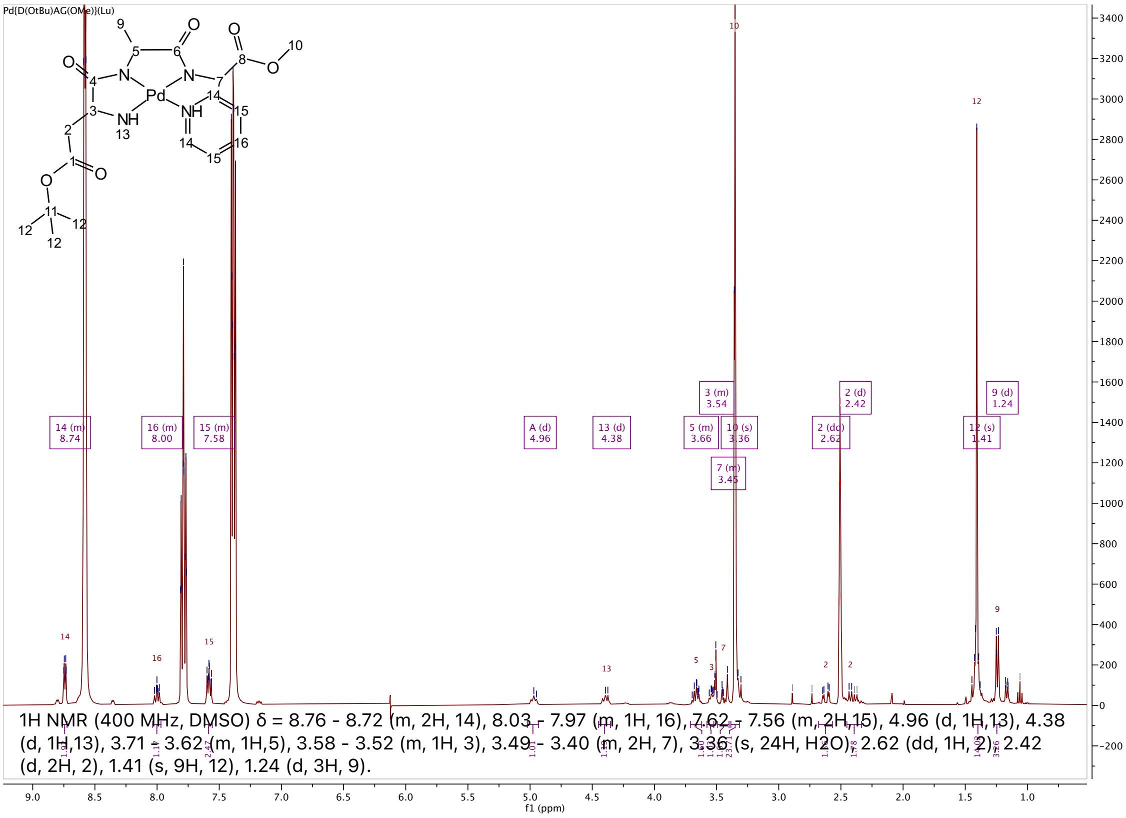

Figure S3a

^{1}H NMR spectra for **8**.

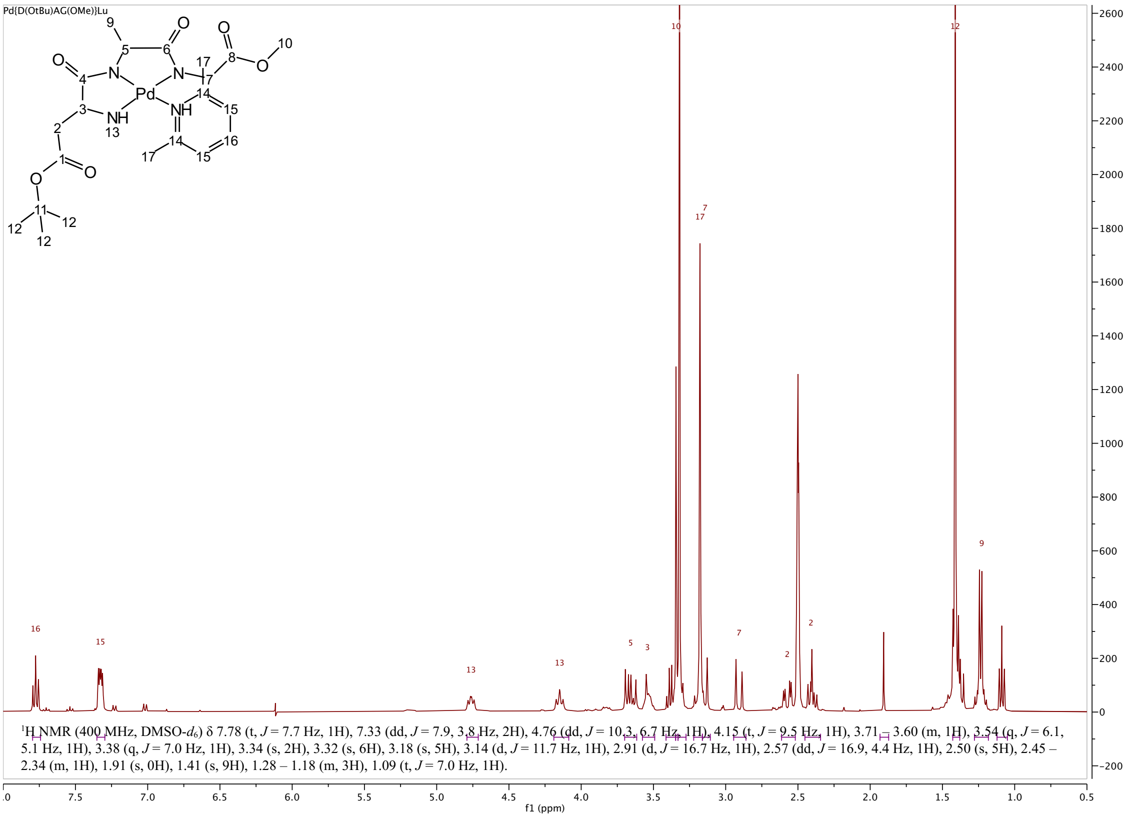

Figure S3b

MS scan for **8**.

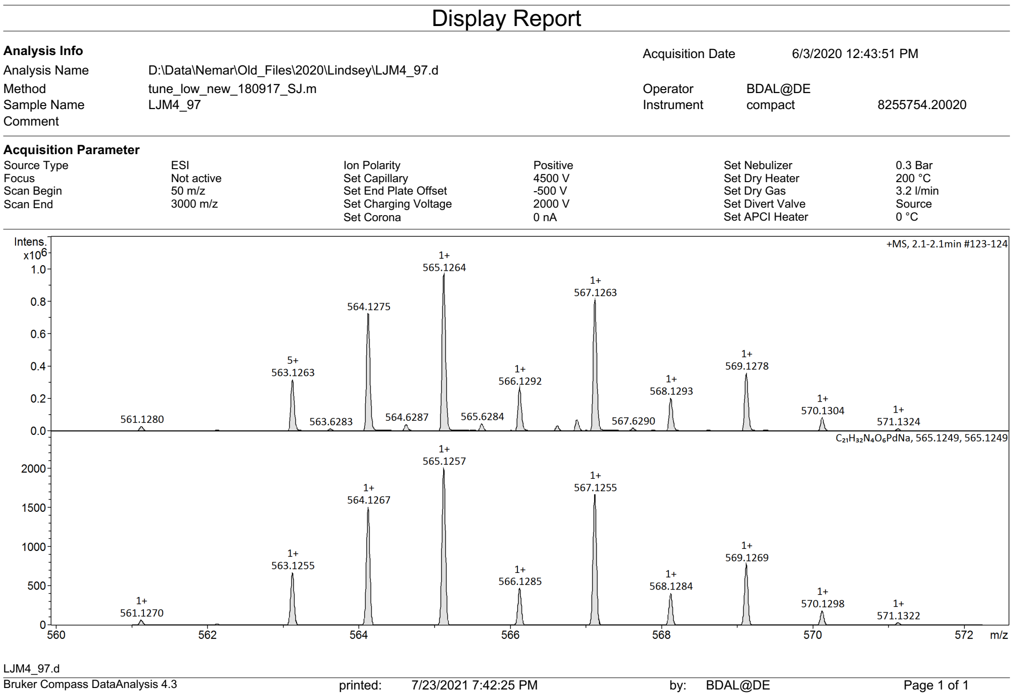

Figure S4a

^{1}H NMR spectra for **9**.

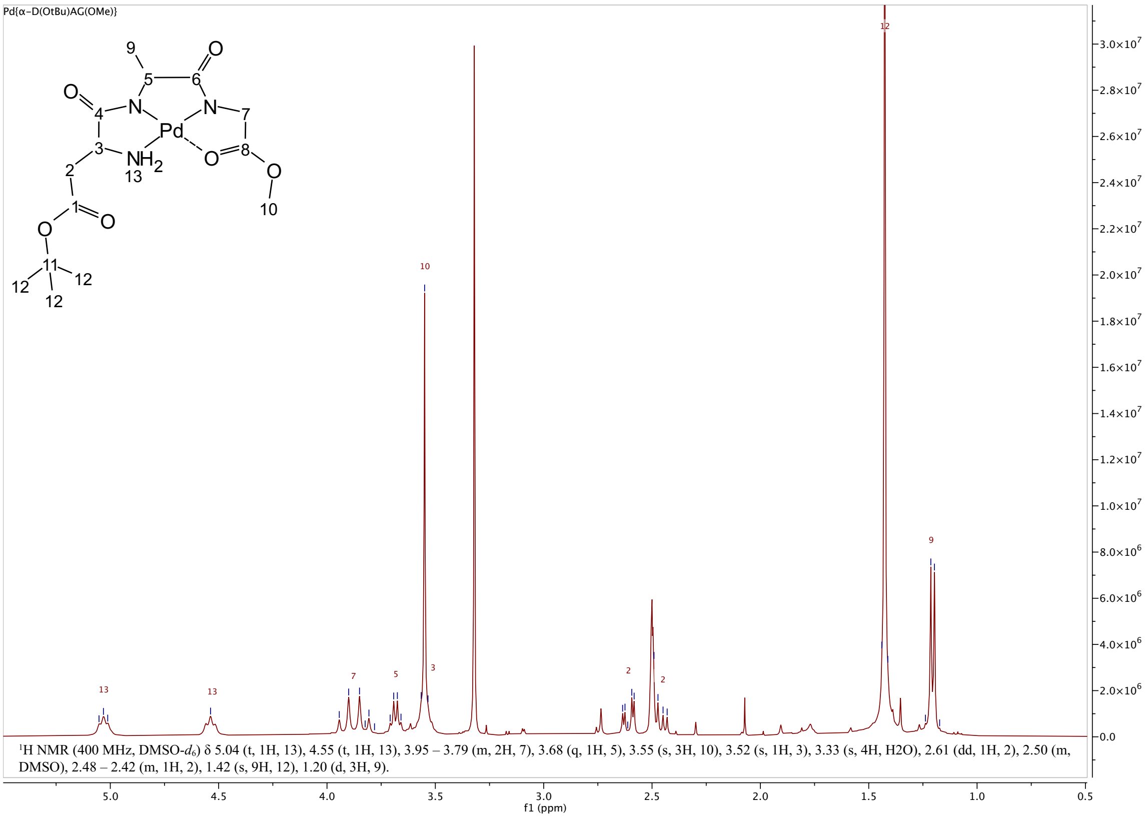

^{1}H NMR (400 MHz, DMSO-d_6) δ 5.04 (t, 1H, 13), 4.55 (t, 1H, 13), 3.95 – 3.79 (m, 2H, 7), 3.68 (q, 1H, 5), 3.55 (s, 3H, 10), 3.52 (s, 1H, 3), 3.33 (s, 4H, H2O), 2.61 (dd, 1H, 2), 2.50 (m, DMSO), 2.48 – 2.42 (m, 1H, 2), 1.42 (s, 9H, 12), 1.20 (d, 3H, 9).

Figure S4b

^{13}C NMR spectra for **9**.

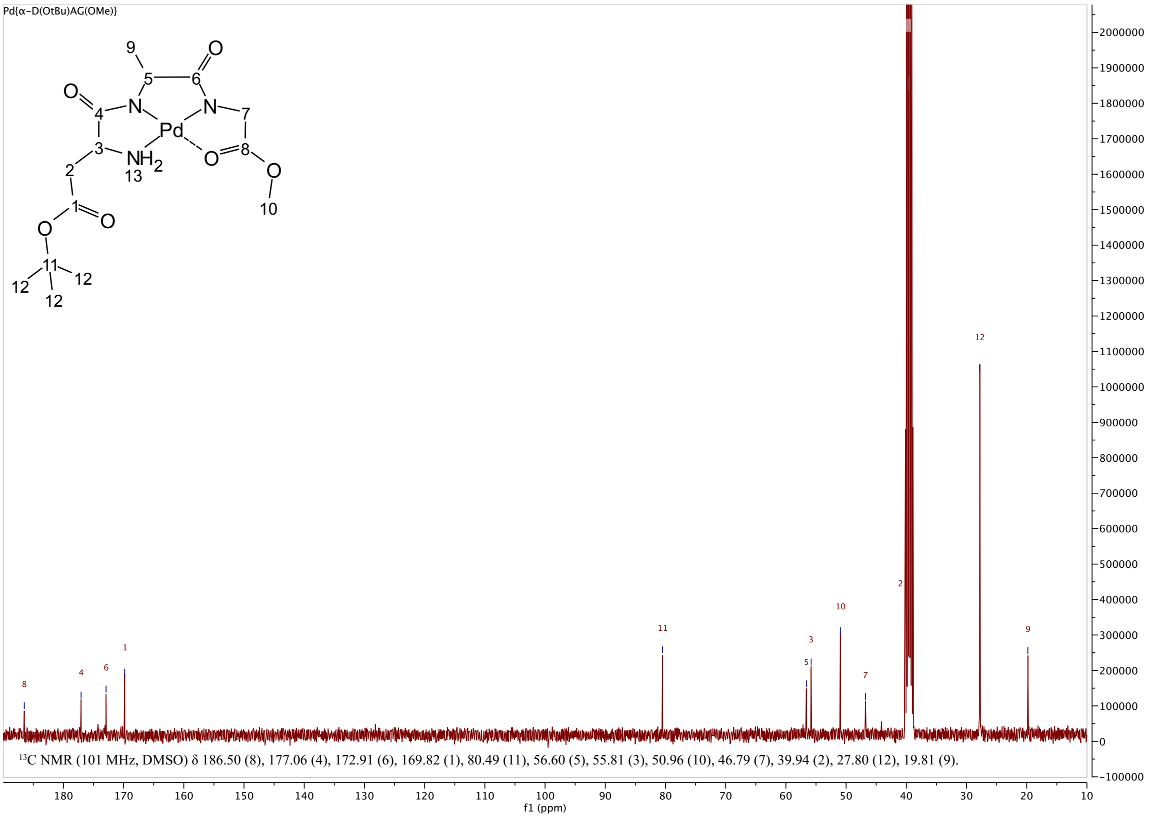

Figure S4c

MS scan for **9**.

Display Report

Analysis Info

Analysis Name	D:\Data\Nemar\Old_Files\2020\Lindsey\LJM4_188_neg.d	Acquisition Date	9/10/2020 11:20:38 AM
Method	tune_low_new_neg_280819_SJ.m	Operator	BDAL@DE
Sample Name	LJM4_188	Instrument	compact 8255754.20020
Comment			

Acquisition Parameter

Source Type	ESI	Ion Polarity	Negative	Set Nebulizer	0.3 Bar
Focus	Not active	Set Capillary	3200 V	Set Dry Heater	200 °C
Scan Begin	50 m/z	Set End Plate Offset	-500 V	Set Dry Gas	3.2 l/min
Scan End	3000 m/z	Set Charging Voltage	2000 V	Set Divert Valve	Source
		Set Corona	0 nA	Set APCI Heater	0 °C

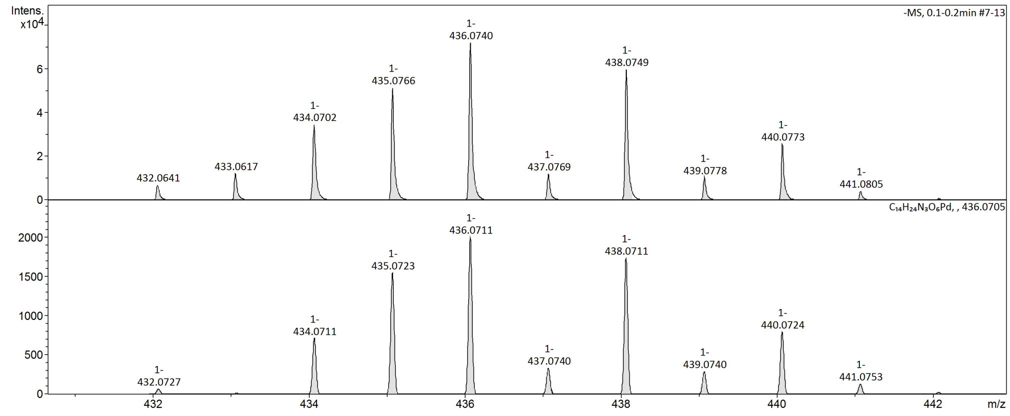

LJM4_188_neg.d

Bruker Compass DataAnalysis 4.3 printed: 7/23/2021 7:41:18 PM by: BDAL@DE Page 1 of 1

Figure S5a

^{1}H NMR spectra for **9** at 330K.

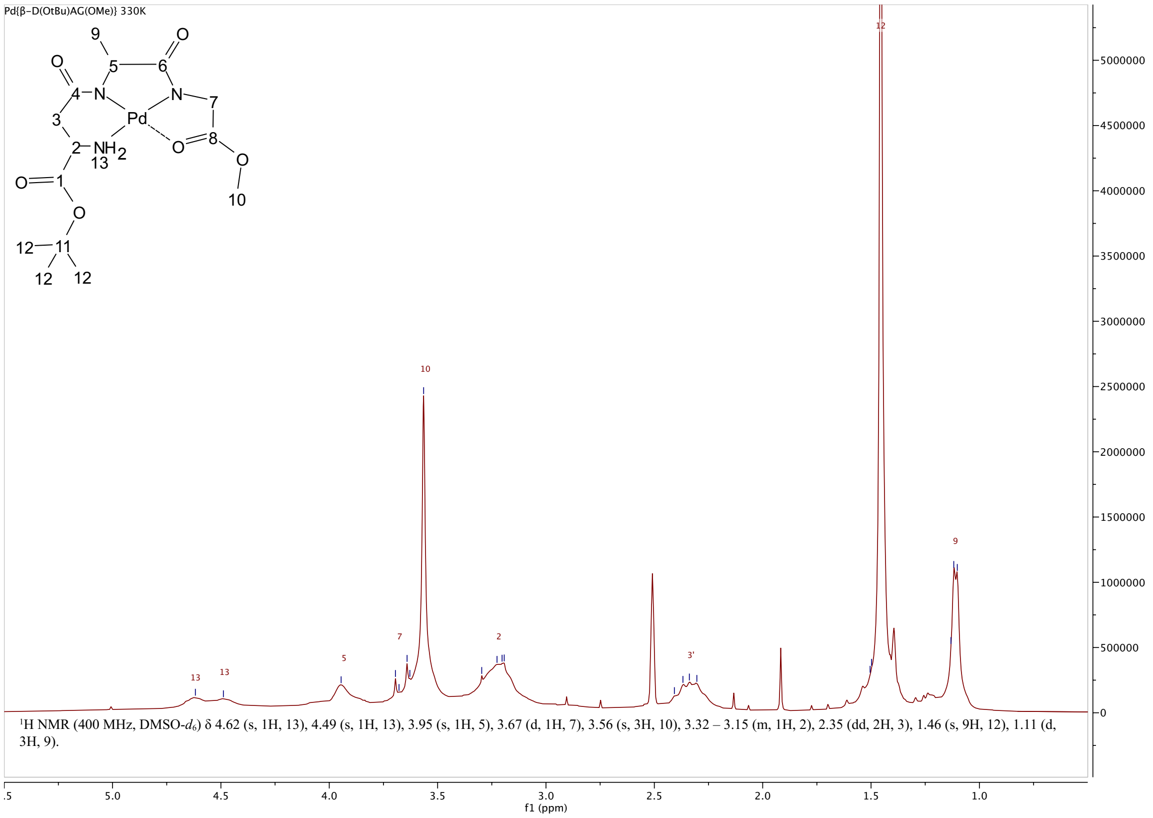

Figure S5b

^{13}C NMR spectra for **9** at 330K.

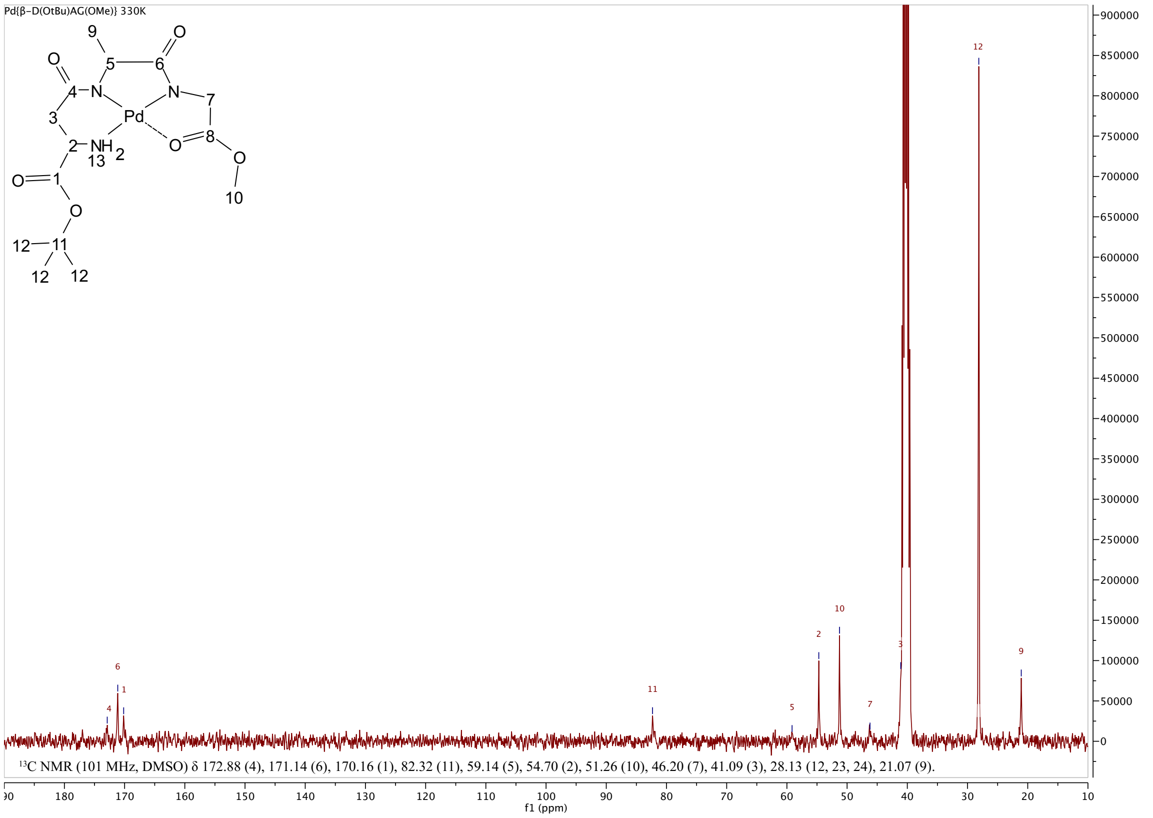

Figure S5c

MS scan for **10**.

Display Report

Analysis Info

Analysis Name	D:\Data\Nemar\Old_Files\2020\Lindsey\LJM4_240.d	Acquisition Date	12/4/2020 2:27:27 PM
Method	tune_low_new_180917_SJ.m	Operator	BDAL@DE
Sample Name	LJM4_240	Instrument	compact 8255754.20020
Comment			

Acquisition Parameter

Source Type	ESI	Ion Polarity	Positive	Set Nebulizer	0.3 Bar
Focus	Not active	Set Capillary	4500 V	Set Dry Heater	200 °C
Scan Begin	50 m/z	Set End Plate Offset	-500 V	Set Dry Gas	3.2 l/min
Scan End	3000 m/z	Set Charging Voltage	2000 V	Set Divert Valve	Source
		Set Corona	0 nA	Set APCI Heater	0 °C

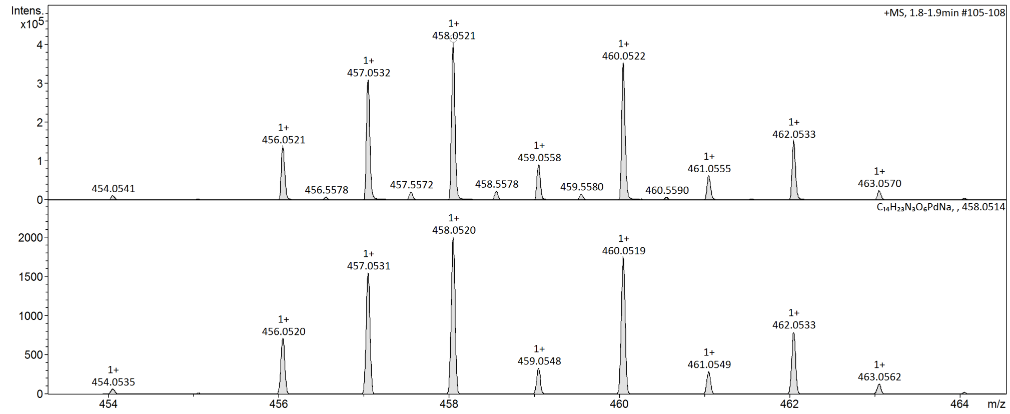

LJM4_240.d

Bruker Compass DataAnalysis 4.3 printed: 7/23/2021 7:37:15 PM by: BDAL@DE Page 1 of 1

Figure S6a

^{1}H NMR spectra for **11**.

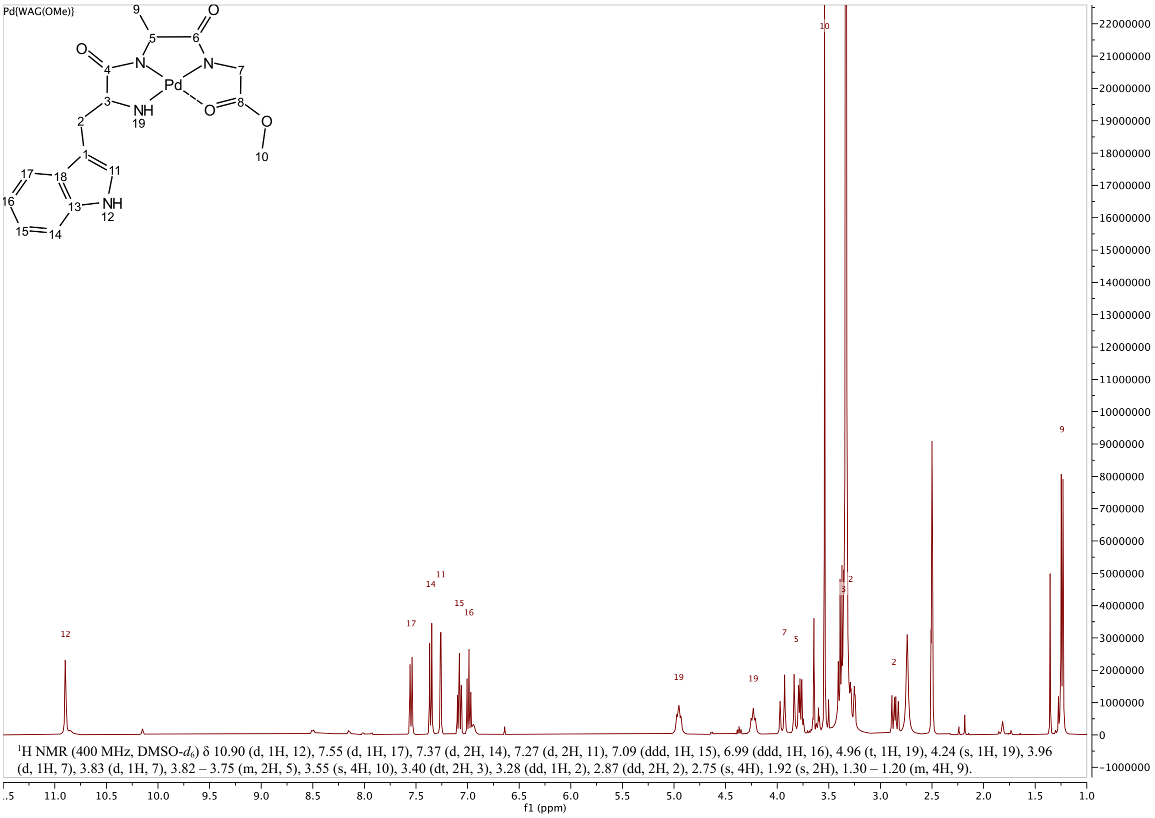

^{1}H NMR (400 MHz, DMSO-d_6) δ 10.90 (d, 1H, 12), 7.55 (d, 1H, 17), 7.37 (d, 2H, 14), 7.27 (d, 2H, 11), 7.09 (ddd, 1H, 15), 6.99 (ddd, 1H, 16), 4.96 (t, 1H, 19), 4.24 (s, 1H, 19), 3.96 (d, 1H, 7), 3.83 (d, 1H, 7), 3.82 – 3.75 (m, 2H, 5), 3.55 (s, 4H, 10), 3.40 (dt, 2H, 3), 3.28 (dd, 1H, 2), 2.87 (dd, 2H, 2), 2.75 (s, 4H), 1.92 (s, 2H), 1.30 – 1.20 (m, 4H, 9).

Figure S6b

^{13}C NMR spectra for **11**.

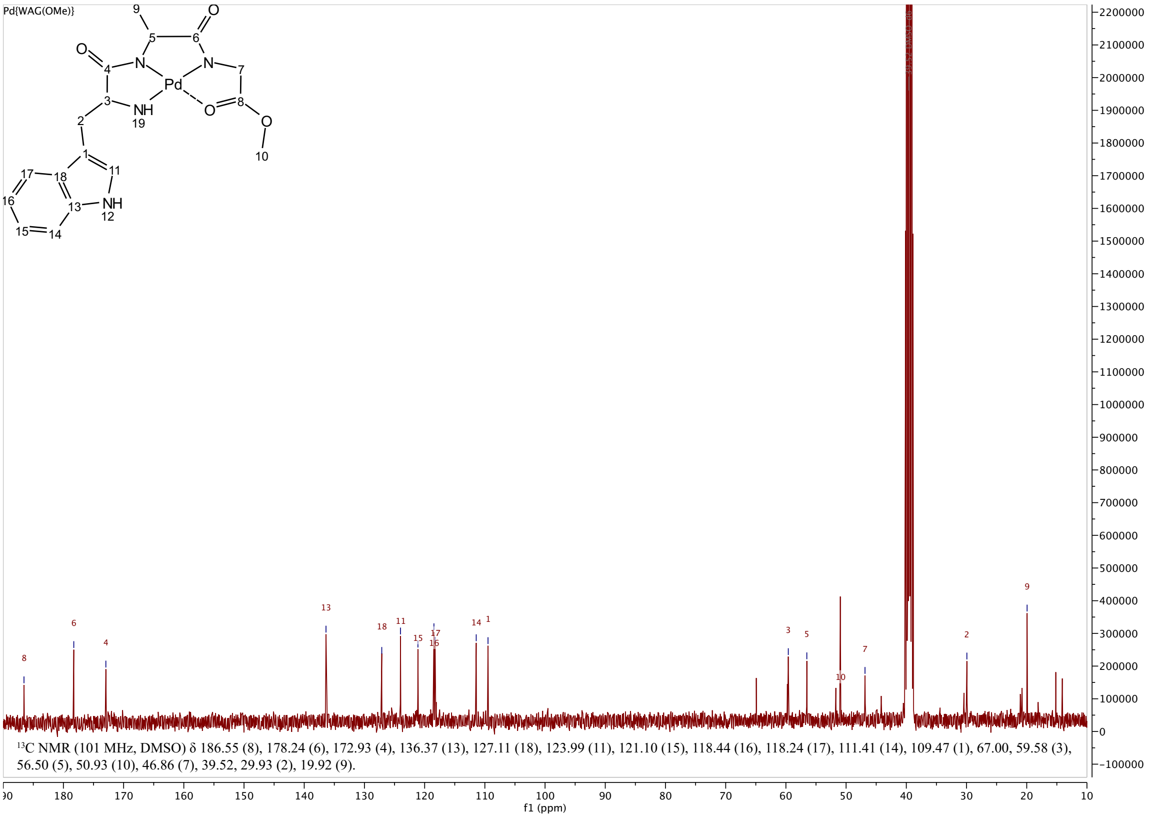

Figure S6c

MS scan for **11**.

Display Report

Analysis Info

Analysis Name	D:\Data\Nemar\Old_Files\2020\Lindsey\LJM4_221.d	Acquisition Date	10/20/2020 11:29:13 AM	
Method	tune_low_new_180917_SJ.m	Operator	BDAL@DE	
Sample Name	LJM4_221	Instrument	compact	8255754.20020
Comment				

Acquisition Parameter

Source Type	ESI	Ion Polarity	Positive	Set Nebulizer	0.3 Bar
Focus	Not active	Set Capillary	4500 V	Set Dry Heater	200 °C
Scan Begin	50 m/z	Set End Plate Offset	-500 V	Set Dry Gas	3.2 l/min
Scan End	3000 m/z	Set Charging Voltage	2000 V	Set Divert Valve	Source
		Set Corona	0 nA	Set APCI Heater	0 °C

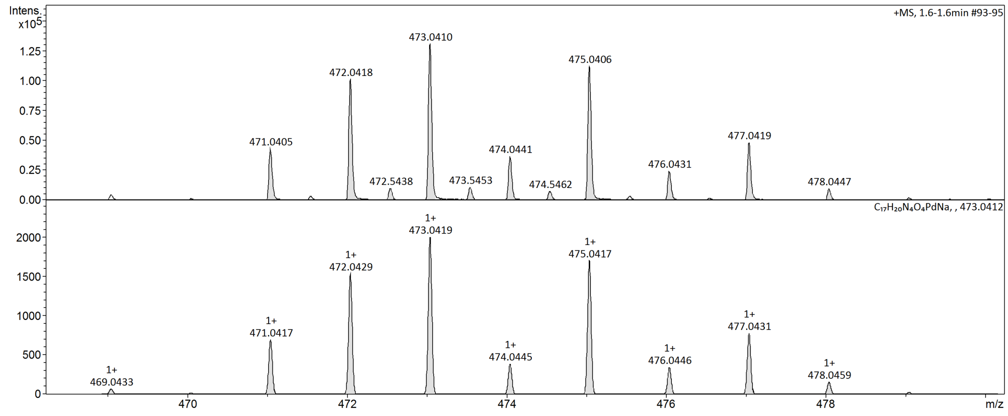

LJM4_221.d

Bruker Compass DataAnalysis 4.3 printed: 7/23/2021 7:39:33 PM by: BDAL@DE Page 1 of 1

Table S1

^{1}H NMR spectra for **1-3**.

Chemical Shift (ppm)	**1**	**2**	**3**	Chemical Shift (ppm)	**1**	**2**	**3**
N—H $_{Trp\text{-}In\ 3}$	—	—	10.86	α-C$_{Ala}$	4.31	4.31	4.34
N—H $_{Gly}$	8.34	8.39	8.32	α-C$_{Gly}$	3.84	3.83	3.83
N—H $_{Ala}$	8.16	8.24	8.12	α-C$_{Trp/Asp}$	3.52	2.49<br>2.36	3.51
C—H $_{Trp\text{-}In\ 7}$	—	—	7.56	OCH_3 $_{Gly}$	3.62	3.63	3.63
C—H $_{Trp\text{-}In\ 4}$	—	—	7.33	β-C$_{Trp/Asp}$	2.37<br>2.35	3.64	3.10<br>2.78
C—H $_{Trp\text{-}In\ 2}$	—	—	7.18	$OC(CH_3)_3$ $_{Asp}$	1.38	1.39	—
C—H $_{Trp\text{-}In\ 5}$	—	—	7.06	β-C$_{Ala}$	1.23	1.21	1.16
C—H $_{Trp\text{-}In\ 6}$	—	—	6.97				

Table S2

^{13}C NMR spectra for **1-3.**

Chemical Shift (ppm)	1	2	3	Chemical Shift (ppm)	1	2	3
C=O$_{Trp/Asp}$	172.82	172.99	173.84	C—H $_{Trp\text{-}In\ 5}$	—	—	111.28
C=O$_{Ala}$	172.61	172.73	172.68	C=C $_{Trp\text{-}In\ 1}$	—	—	110.22
γ-C=O$_{Asp}$	170.45	170.17	—	$O\underline{C}(CH_3)_3$ $_{Asp}$	79.97	80.41	—
C=O$_{Gly}$	170.11	169.4	170.12	OCH_3 $_{Gly}$	51.68	51.67	52.69
C=C $_{Trp\text{-}In\ 4}$	—	—	136.20	α-C$_{Trp/Asp}$	51.51	39.15	54.91
C=C $_{Trp\text{-}In\ 9}$	—	—	127.39	α-C$_{Ala}$	47.78	47.85	47.52
C—H $_{Trp\text{-}In\ 2}$	—	—	123.94	α-C$_{Gly}$	40.48	40.48	40.48
C—H $_{Trp\text{-}In\ 6}$	—	—	120.84	β-C$_{Trp/Asp}$	40.24	51.52	30.42
C—H $_{Trp\text{-}In\ 8}$	—	—	118.46	$OC(\underline{C}H_3)_3$ $_{Asp}$	27.75	27.61	—
C—H $_{Trp\text{-}In\ 7}$	—	—	118.19	β-C$_{Ala}$	18.50	18.18	18.64

Table S3
Select functional group IR vibrations for **1-3**

Frequency (cm^{-1})	**1**	**2**	**3**
ν(N—H_2)	3370	3375*	3370*
ν(N—H)	3314	3304	3297
ν(C=O)			
OtBu	1725	1743	—
OMe	1759*	1757*	1749
Amide I	1670	1659	1655
ν(C=C)			
Aromatic	—	—	1673*
ν(N—H)			
Amide II	1546	1541	1517
ν(C—O)			
OtBu	1250	1254	—
OMe	1213	1212	1213
ν(O—C)			
OtBu	1155	1155	—

Figure S7
Stacked IR spectra for **1**, **4**, **8**, and **9**.

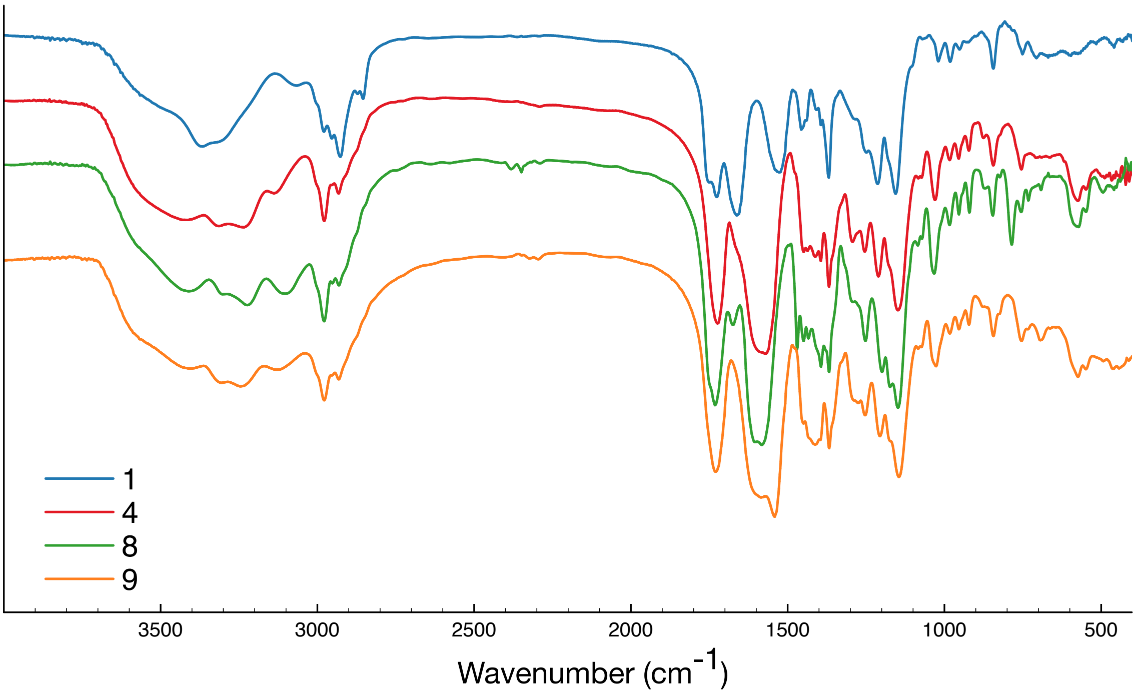

Figure S8

^{1}H NMR spectra for **10** in DMSO-*d*6: 1) before and 3) after catalytic sulfur transfer reactions. Compared to 2) free ligand **2**. NMR suggests complex is demetallated.

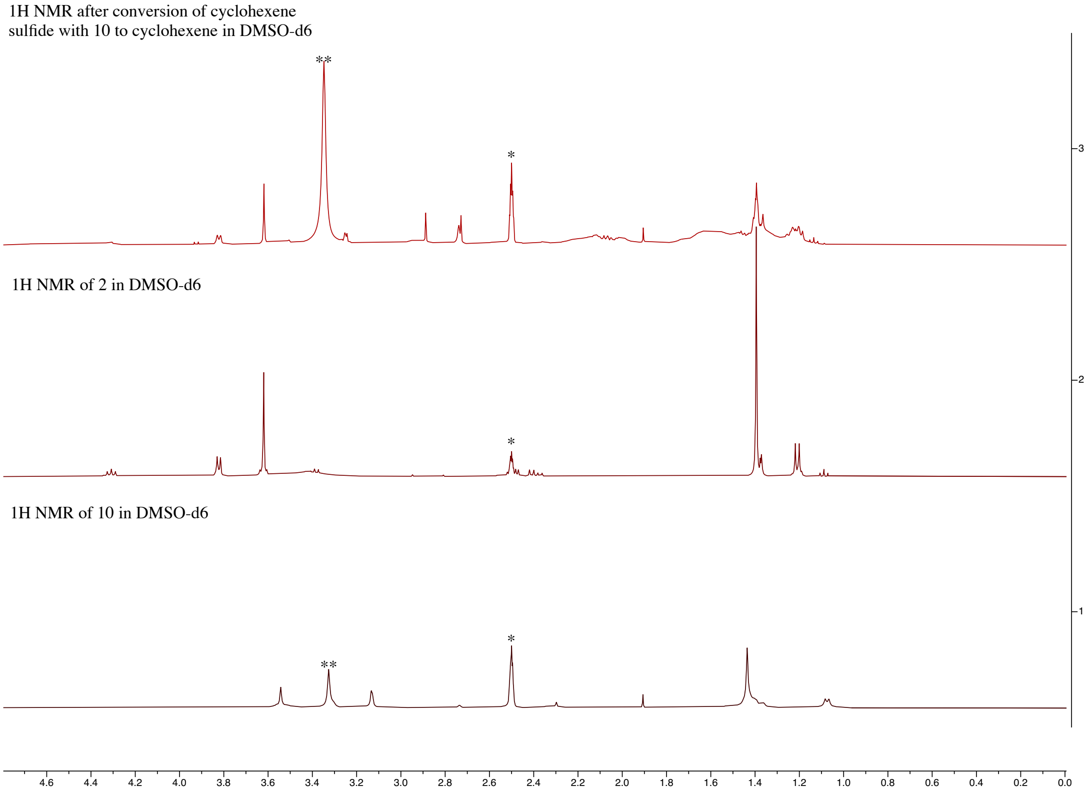

Figure S9

Example of reaction progress for cyclohexene sulfide SAT by **10** in dmso-*d*6

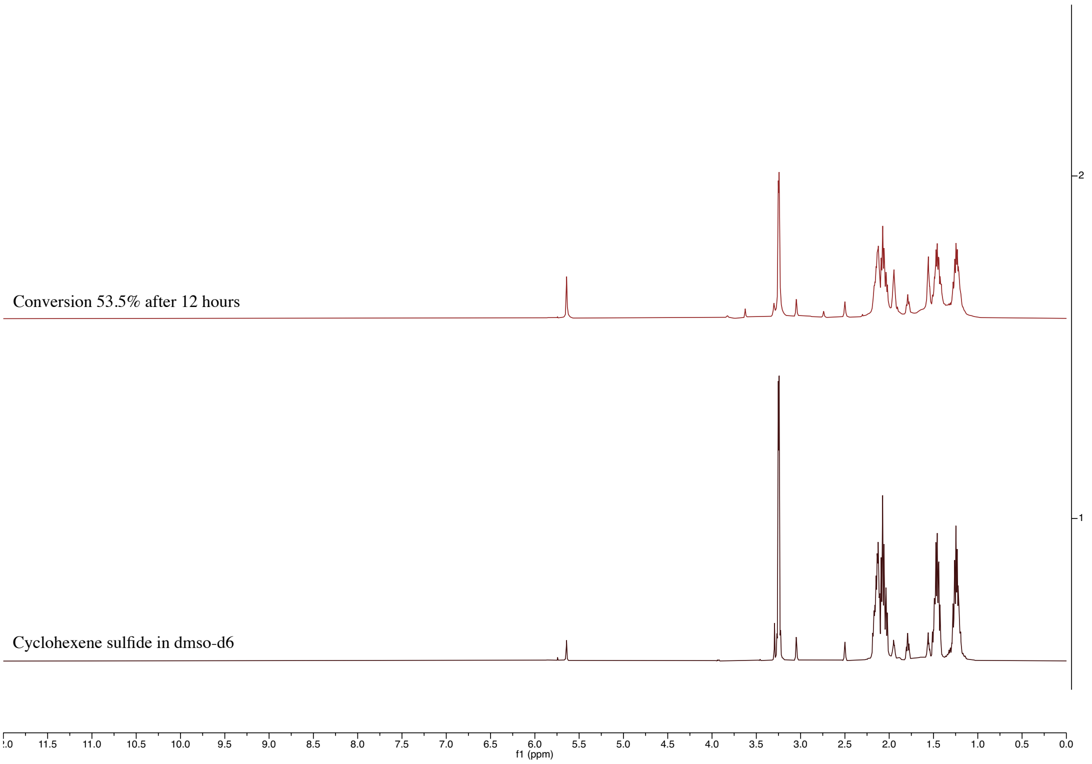

Figure S10

Stacked ^{1}H NMR spectra of **9** (1) with addition of: (2) 30 µL TFA, (3) Ethylene, (4) 20 µL TFA and Ethylene, (5) 30 µL TFA and Ethylene, (6) 40 µL TFA and Ethylene, (7) 40 µL TFMS and Ethylene, (8) Ligand 1, (9) Blank with Ethylene and TFA

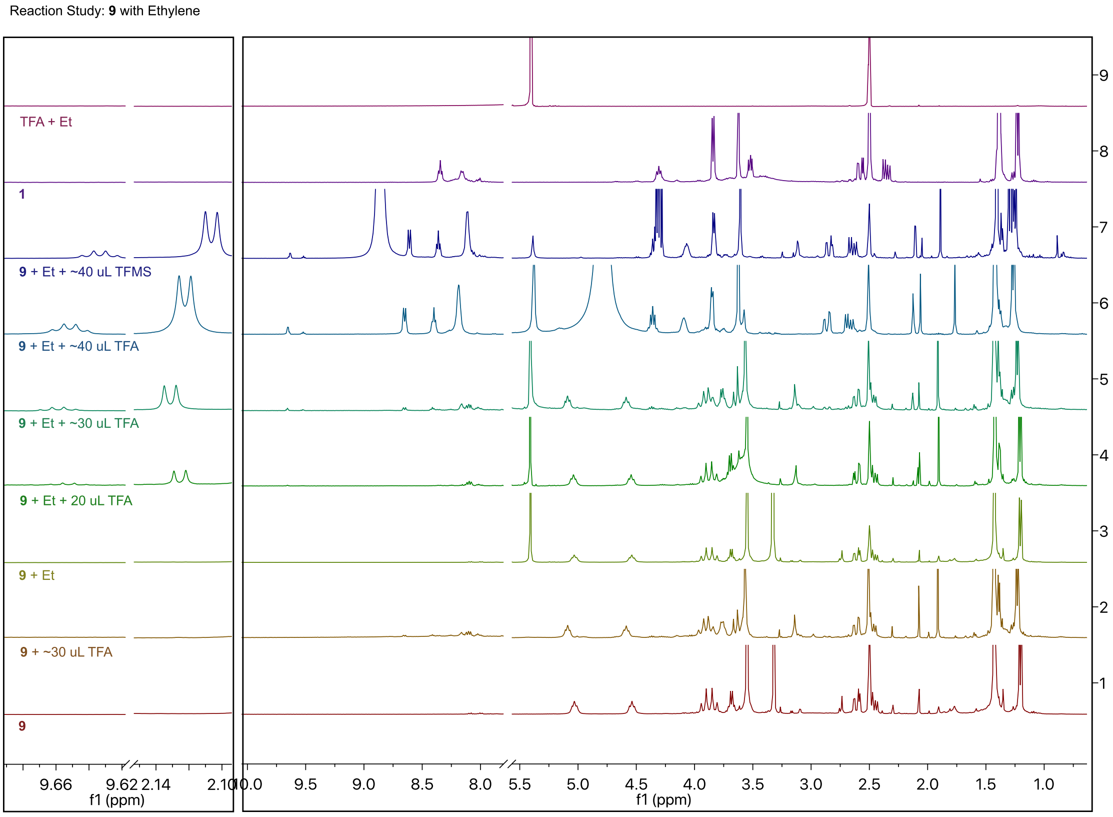

Cartesian coordinates for complexes in angstrom

Complex **4** K[Pd{D(OtBu)AG(OMe)}Cl]
C -3.4816210000 4.1614410000 -5.0538940000
O -3.4680930000 4.9026440000 -6.0425390000
N -4.2856640000 3.0883840000 -4.9172690000
C -5.1781560000 2.8108550000 -6.0116850000
C -6.2849050000 3.8356920000 -6.1998100000
O -6.7173730000 4.6110550000 -5.3914390000
Pd -4.1316070000 2.0732380000 -3.1955380000
N -2.7122060000 3.4069630000 -2.8579500000
C -2.0758500000 3.3902390000 -1.6914450000
O -1.2565830000 4.2069150000 -1.2479010000
C -2.3578350000 2.1063980000 -0.8642370000
N -3.6647880000 1.4925440000 -1.2315410000
H -3.6841060000 0.4939950000 -1.0511460000
H -4.4106760000 1.8960480000 -0.6739420000
C -1.2082820000 1.1414760000 -1.1403240000
C -1.1803560000 -0.0951590000 -0.2711610000
O -1.9750460000 -0.3517650000 0.6035780000
O -0.1400700000 -0.8767870000 -0.6165960000
C 0.1378700000 -2.1645770000 0.0371550000
C -1.0348620000 -3.1240140000 -0.1650870000
H -1.9172410000 -2.7934940000 0.3774960000
H -1.2832100000 -3.2012400000 -1.2251410000
H -0.7562990000 -4.1183420000 0.1912650000
C 1.3731790000 -2.6589380000 -0.7131140000
H 1.6940050000 -3.6231990000 -0.3144330000
H 1.1549110000 -2.7766360000 -1.7752960000
H 2.1943350000 -1.9481700000 -0.6088740000
C 0.4589130000 -1.9417570000 1.5150610000
H 1.2598720000 -1.2075430000 1.6221750000
H -0.4140090000 -1.5909950000 2.0610380000
H 0.7976510000 -2.8804760000 1.9594730000
H -1.2280240000 0.8283460000 -2.1882950000
H -0.2673530000 1.6783140000 -0.9985380000
H -2.3509680000 2.3780740000 0.1930400000
C -2.5212670000 4.4279590000 -3.8777190000
C -1.0798020000 4.4956300000 -4.3933910000
H -0.4024610000 4.7718200000 -3.5865450000
H -0.7781710000 3.5233360000 -4.7924450000
H -1.0231060000 5.2305380000 -5.1969830000
H -2.7899900000 5.4118560000 -3.4735690000
O -6.8039410000 3.7274320000 -7.4583270000
C -7.8881250000 4.6071890000 -7.7507880000
H -7.5729910000 5.6488310000 -7.6719630000
H -8.7214240000 4.4428510000 -7.0654160000
H -8.1889430000 4.3807130000 -8.7725010000
H -4.6433570000 2.7366770000 -6.9634830000
H -5.6678680000 1.8526160000 -5.8216210000
Cl -5.7849020000 0.3883790000 -3.4884210000

Complex **8** Pd{D(OtBu)AG(OMe)}Lu
C -3.6395860000 4.0574120000 -5.1160890000
O -3.7452220000 4.7593320000 -6.1191620000
N -4.4724110000 3.0205900000 -4.8422290000
C -5.4582240000 2.7493730000 -5.8664200000
C -6.5178830000 3.8368040000 -6.0094810000
O -7.0182250000 4.4496770000 -5.0981650000
Pd -4.2055590000 2.1438680000 -3.0560590000
N -2.6308260000 3.3193700000 -3.0243350000
C -1.8294300000 3.3140420000 -1.9536580000
O -0.9120870000 4.0909990000 -1.6950140000
C -2.0822620000 2.1039030000 -1.0245330000
N -3.4994670000 1.6410740000 -1.1195940000
H -3.5430670000 0.6638310000 -0.8295510000
H -4.0619450000 2.1728470000 -0.4628330000
C -1.1033920000 0.9974470000 -1.4394330000
C -1.2059790000 -0.2900570000 -0.6490950000
O -2.2631930000 -0.7766290000 -0.2927730000
O -0.0082960000 -0.8300220000 -0.4432690000
C 0.1869900000 -2.1469530000 0.2150500000
C -0.4931260000 -3.2398380000 -0.6059570000
H -1.5760200000 -3.1400100000 -0.5868820000
H -0.1498560000 -3.2089660000 -1.6417210000
H -0.2283730000 -4.2152630000 -0.1935300000
C 1.7036100000 -2.3071520000 0.1829540000
H 1.9863800000 -3.2559460000 0.6411980000
H 2.0716040000 -2.2954980000 -0.8436790000
H 2.1887470000 -1.4993480000 0.7319620000
C -0.3254270000 -2.0899410000 1.6517100000
H 0.1263920000 -1.2518130000 2.1852130000
H -1.4078180000 -1.9907890000 1.6864960000
H -0.0434170000 -3.0091330000 2.1689150000
H -1.2894590000 0.7376040000 -2.4876420000
H -0.0916890000 1.3936150000 -1.3819760000
H -1.8607150000 2.4154780000 -0.0015480000
C -2.5096380000 4.2910550000 -4.1042700000
C -1.1582900000 4.2256600000 -4.8240160000
H -0.3522560000 4.4704900000 -4.1352430000
H -0.9908550000 3.2232690000 -5.2262290000
H -1.1622320000 4.9334360000 -5.6528200000
H -2.6414760000 5.3034990000 -3.7055260000
O -6.8880040000 3.9863300000 -7.2927930000
C -7.8792970000 4.9915830000 -7.5494940000
H -7.5104410000 5.9698390000 -7.2426610000
H -8.8014790000 4.7723840000 -7.0108180000
H -8.0489000000 4.9684040000 -8.6229330000
H -4.9869930000 2.6146660000 -6.8424610000
H -5.9885010000 1.8268710000 -5.6193300000
C -8.1916270000 -0.7225240000 -2.9909590000
C -8.2573290000 0.5900680000 -2.5576950000
C -7.1185370000 1.3913910000 -2.5961830000
N -5.9432040000 0.8979630000 -3.0503610000
C -5.8675000000 -0.3817100000 -3.4791200000
C -6.9832710000 -1.2120040000 -3.4587860000
H -9.0701020000 -1.3552560000 -2.9665820000
H -9.1838840000 1.0096210000 -2.1909910000

H -6.8930230000 -2.2308260000 -3.8095650000
C -7.1787790000 2.8217400000 -2.1557240000
H -6.4262970000 3.0322350000 -1.3939100000
H -6.9846740000 3.4918600000 -2.9977710000
H -8.1620810000 3.0516030000 -1.7465280000
C -4.5456660000 -0.8912660000 -3.9725100000
H -3.8418730000 -0.9986060000 -3.1435880000
H -4.6625250000 -1.8650200000 -4.4473670000
H -4.1015770000 -0.1959660000 -4.6856910000

Complex **9** Pd{D(OtBu)AG(OMe)}
C 1.1012480000 0.6289550000 -1.3674620000
O 1.0472260000 1.3790620000 -2.3339320000
N 0.4065610000 -0.5242210000 -1.2568450000
C -0.5606400000 -0.9316110000 -2.2369150000
C -1.3348410000 -2.0903430000 -1.6633120000
O -1.1589670000 -2.5444570000 -0.5318330000
Pd 0.4153320000 -1.4047330000 0.4841640000
N 1.7776550000 -0.0868790000 0.8876970000
C 2.3553190000 -0.0808430000 2.0934050000
O 3.1403210000 0.7509960000 2.5391180000
C 2.0248370000 -1.3515340000 2.9069500000
N 0.6972110000 -1.9158620000 2.5178630000
H 0.6660970000 -2.8897430000 2.8166860000
H -0.0314130000 -1.4271610000 3.0287990000
C 3.1398930000 -2.3591830000 2.6294120000
C 3.0018310000 -3.6825190000 3.3462370000
O 1.9447110000 -4.1395610000 3.7333780000
O 4.1816090000 -4.2859720000 3.4502230000
C 4.3297660000 -5.6546790000 3.9971370000
C 3.5471210000 -6.6374110000 3.1337280000
H 2.4752840000 -6.4757500000 3.2166420000
H 3.8431180000 -6.5421520000 2.0877120000
H 3.7715800000 -7.6551570000 3.4571150000
C 5.8281330000 -5.8979820000 3.8750200000
H 6.0726590000 -6.8932760000 4.2479050000
H 6.1453210000 -5.8295700000 2.8340550000
H 6.3852210000 -5.1624660000 4.4559280000
C 3.8906330000 -5.6794030000 5.4565570000
H 4.4137760000 -4.9075210000 6.0230460000
H 2.8189210000 -5.5258400000 5.5526480000
H 4.1456370000 -6.6483120000 5.8894550000
H 3.1659330000 -2.5789730000 1.5564720000
H 4.0970750000 -1.9040170000 2.8783120000
H 2.0257890000 -1.0886540000 3.9659250000
C 2.0154150000 0.9122820000 -0.1560000000
C 3.4738920000 0.9385980000 -0.6116550000
H 4.1207920000 1.2136450000 0.2188550000
H 3.7671570000 -0.0466070000 -0.9811710000
H 3.5844600000 1.6618140000 -1.4195080000
H 1.7425570000 1.9030230000 0.2210410000
O -2.2363140000 -2.5939110000 -2.4838480000
C -3.0170530000 -3.7072990000 -2.0002380000
H -3.5733050000 -3.4153920000 -1.1112280000
H -2.3647080000 -4.5461430000 -1.7636340000
H -3.6907410000 -3.9577570000 -2.8134300000
H -1.2657760000 -0.1324940000 -2.4992580000
H -0.1101910000 -1.2540980000 -3.1842120000

Complex **10** Pd{iso-D(OtBu)AG(OMe)}
C -0.8652210000 2.9869730000 -1.6592760000
O -1.2287870000 3.7060370000 -2.5787770000
N -1.4681940000 2.9212960000 -0.4622610000
C -2.7887960000 3.4697420000 -0.2879850000
C -3.5939580000 2.5053330000 0.5452020000
O -3.1364300000 1.4851260000 1.0499120000
O -4.8505290000 2.8673010000 0.7241700000
C -5.6646430000 2.0208020000 1.5319630000
H -5.2496410000 1.9439010000 2.5386990000
H -6.6462320000 2.4856900000 1.5582540000
H -5.7258410000 1.0228210000 1.0945810000
H -2.7773220000 4.4415450000 0.2309150000
H -3.2682410000 3.6320900000 -1.2656000000
C 0.2901930000 1.9772490000 -1.7765070000
N 0.6924110000 1.5443370000 -0.4473180000
C 1.7870140000 0.8121970000 -0.2917510000
O 2.5976860000 0.5355190000 -1.1685780000
C 2.1012800000 0.4089090000 1.1753160000
H 2.2641060000 1.3484950000 1.7060590000
C 1.0552960000 -0.4412810000 1.9282100000
C 0.6158380000 0.2490480000 3.2299950000
O 0.5996060000 -0.2931040000 4.3037940000
O 0.2251050000 1.4772780000 2.9458660000
C -0.4277250000 2.3508820000 3.9078580000
C -0.6341980000 3.6373610000 3.1070010000
H 0.3085410000 4.1652790000 2.9896250000
H -1.3481120000 4.2894410000 3.6010220000
H -1.0008890000 3.3700090000 2.1046330000
C 0.4601730000 2.5792800000 5.1263130000
H 0.0018780000 3.3225770000 5.7725840000
H 1.4370930000 2.9413340000 4.8143070000
H 0.5914970000 1.6573640000 5.6859450000
C -1.7668880000 1.7165320000 4.2805120000
H -2.3517020000 2.3994650000 4.8898760000
H -2.3183300000 1.4853700000 3.3665530000
H -1.6080120000 0.7977070000 4.8400540000
N -0.1861140000 -0.6363610000 1.1533570000
H 0.0466710000 -1.1150800000 0.2774320000
H -0.8220540000 -1.2371370000 1.6810370000
H 1.4890070000 -1.4151170000 2.1996310000
H 3.0489080000 -0.1291620000 1.1410830000
H 1.1438250000 2.4048620000 -2.3143810000
C -0.2728350000 0.7826900000 -2.5694920000
H -1.0977690000 0.3209670000 -1.9950580000
H 0.5163350000 0.0511700000 -2.7311650000
H -0.6538940000 1.1209370000 -3.5297760000
Pd -0.9696520000 1.2973160000 0.5165770000

Complex **11** Pd{WAG(OMe)}
C -0.7382300000 6.0774160000 -3.7259970000
O -1.5112880000 5.9553040000 -4.6691070000
N 0.6034930000 6.1654740000 -3.8467360000
C 1.2745630000 6.1188890000 -5.1164690000
C 2.7530030000 6.2728250000 -4.8561750000
O 3.2446490000 6.3984080000 -3.7338360000
O 3.4951280000 6.2610530000 -5.9477550000
C 4.9225330000 6.4041990000 -5.7834840000
H 5.1509920000 7.3513450000 -5.2977700000
H 5.3289880000 6.3788710000 -6.7897920000
H 5.3154990000 5.5825430000 -5.1869160000
H 1.1141750000 5.1766510000 -5.6573360000
H 0.9553690000 6.9127120000 -5.8042160000
C -1.2306800000 6.1683500000 -2.2615020000
N -0.0813020000 6.2538800000 -1.3530500000
C -0.1493120000 6.0242230000 -0.0382440000
O -1.1725050000 5.8160600000 0.6123280000
C 1.2329790000 5.9711700000 0.6600720000
N 2.3026330000 6.5659540000 -0.2032060000
H 3.2046160000 6.1610300000 0.0247390000
H 2.3697220000 7.5630380000 -0.0055810000
H 1.4662740000 4.9060700000 0.7462140000
H -1.7767500000 5.2426800000 -2.0510310000
C -2.1957730000 7.3464120000 -2.1097820000
H -2.6204160000 7.3534710000 -1.1069570000
H -1.6751280000 8.2908910000 -2.2836220000
H -2.9979340000 7.2513170000 -2.8422810000
C 1.1883780000 6.5784030000 2.0709460000
C 1.0136670000 8.0685740000 2.0911970000
H 2.1022050000 6.3046120000 2.6064670000
H 0.3585130000 6.0928150000 2.5858730000
C -0.1699510000 8.7481570000 1.9676130000
N 0.0602160000 10.1043740000 2.0040310000
C 1.4065940000 10.3370860000 2.1507420000
C 2.0436750000 9.0692410000 2.2111900000
H -1.1652420000 8.3523940000 1.8464850000
C 2.1169530000 11.5326870000 2.2394370000
C 3.4367450000 9.0269000000 2.3779830000
C 4.1447080000 10.2131170000 2.4654910000
C 3.4910410000 11.4550290000 2.3938840000
H -0.6497350000 10.8107270000 1.9300910000
H 1.6143320000 12.4912680000 2.1912500000
H 4.0709230000 12.3664560000 2.4647900000
H 3.9570670000 8.0786610000 2.4505180000
H 5.2190860000 10.1880410000 2.5978870000
